# Zbtb16 mediates a switch between Fgf signalling regimes in the developing hindbrain

**DOI:** 10.1242/dev.201319

**Published:** 2023-09-14

**Authors:** Sami A. Leino, Sean C. J. Constable, Andrea Streit, David G. Wilkinson

**Affiliations:** ^1^ Neural Development Laboratory, The Francis Crick Institute, 1 Midland Road, London NW1 1AT, UK; ^2^ Centre for Craniofacial and Regenerative Biology, Faculty of Dentistry, Oral and Craniofacial Sciences, King's College London, London SE1 1UL, UK

**Keywords:** Zbtb16, Hindbrain, Fgf signalling, Neurogenesis, Zebrafish

## Abstract

Developing tissues are sequentially patterned by extracellular signals that are turned on and off at specific times. In the zebrafish hindbrain, fibroblast growth factor (Fgf) signalling has different roles at different developmental stages: in the early hindbrain, transient Fgf3 and Fgf8 signalling from rhombomere 4 is required for correct segmentation, whereas later, neuronal Fgf20 expression confines neurogenesis to specific spatial domains within each rhombomere. How the switch between these two signalling regimes is coordinated is not known. We present evidence that the Zbtb16 transcription factor is required for this transition to happen in an orderly fashion. Zbtb16 expression is high in the early anterior hindbrain, then gradually upregulated posteriorly and confined to neural progenitors. In mutants lacking functional Zbtb16, *fgf3* expression fails to be downregulated and persists until a late stage, resulting in excess and more widespread Fgf signalling during neurogenesis. Accordingly, the spatial pattern of neurogenesis is disrupted in Zbtb16 mutants. Our results reveal how the distinct stage-specific roles of Fgf signalling are coordinated in the zebrafish hindbrain.

## Teaser

**Summary:** Dynamic expression of Zbtb16 underlies downregulation of early segmental *fgf3* expression in the zebrafish hindbrain, enabling the correct pattern of Fgf signalling at later stages when Fgf20 organizes neurogenesis.

## INTRODUCTION

During development of the vertebrate central nervous system (CNS), the hindbrain is transiently subdivided into seven or eight segments called rhombomeres along its antero-posterior axis. This segmental organization underlies the generation of the various neuronal subtypes that arise from the hindbrain (Lumsden and Keynes, 1989; Marin and Puelles, 1995). Each rhombomere acquires a distinct identity on the basis of intercellular signalling and gene regulatory interactions (reviewed by Frank and Sela-Donenfeld, 2019; Krumlauf and Wilkinson, 2021), which in turn underlie the identity of hindbrain-derived neurons (reviewed by Philippidou and Dasen, 2013). After the demarcation of segmental identities, rhombomere boundaries (the interfaces between two adjacent rhombomeres) acquire properties distinct from rhombomere centres (Lumsden and Keynes, 1989; Trevarrow et al., 1990; Guthrie and Lumsden, 1991; Heyman et al., 1995). In zebrafish, hindbrain boundaries are induced at segmental interfaces by a process involving mechanical tension (Cayuso et al., 2019) and in turn regulate the spatial pattern of neuronal differentiation by local inhibition of neurogenesis (Cheng et al., 2004; Voltes et al., 2019) and by signalling to non-boundary populations (Riley et al., 2004; Gonzalez-Quevedo et al., 2010; Terriente et al., 2012).

Fgf signalling has different functions in the hindbrain depending on the developmental stage. In zebrafish, rhombomere (r) 4 acts as a signalling centre during early hindbrain development: *fgf8* (also known as *fgf8a*), and later *fgf3*, are expressed in r4 from late gastrulation until mid-somitogenesis and regulate gene expression in r3 and r5, pointing to a role for Fgf signalling in regulating rhombomere identity (Maves et al., 2002; Walshe et al., 2002; Wiellette and Sive, 2004). At later stages, after downregulation of segmental *fgf8* and *fgf3* expression, Fgf20 signalling from neurons located in rhombomere centres antagonizes neurogenesis and may have a role in promoting gliogenesis (Gonzalez-Quevedo et al., 2010; Esain et al., 2010; Tambalo et al., 2020). As neuronal differentiation is also inhibited at rhombomere boundaries (Cheng et al., 2004; Voltes et al., 2019), neurogenesis only takes place in stripes between rhombomere centres and boundaries. Thus, in the zebrafish hindbrain, early Fgf signalling regulates rhombomere identity, whereas late Fgf signalling establishes the spatial pattern of neuronal differentiation, and these two functions involve different ligands. This change is reflected in the expression pattern of the Fgf target gene *etv5b*, which is expressed first in a rhombomere-specific pattern, and later confined to segment centres (Münchberg et al., 1999; Roehl and Nüsslein-Volhard, 2001; Esain et al., 2010; Gonzalez-Quevedo et al., 2010).

In amniotes, the early patterning of rhombomeres is also regulated by Fgf signalling. In chick, the expression of r3-r6 markers is dependent on Fgf receptor signalling (Marin and Charnay, 2000; Aragon et al., 2005; Aragon and Pujades, 2009) and the Fgf3 ligand specifically (Weisinger et al., 2008, 2010). Fgf3 itself is initially expressed in a dynamic, segment-specific pattern and is later restricted to rhombomere boundaries in chick (Mahmood et al., 1995; Weisinger et al., 2008, 2010, 2012; Sela-Donenfeld et al., 2009) and mouse (Wilkinson et al., 1988; Mahmood et al., 1996). In the chick hindbrain, rhombomere boundaries act as pools of neural progenitors (Peretz et al., 2016) and the expression of neuronal markers at the boundaries is dependent on Fgf3 function (Weisinger et al., 2012). These observations suggest that, in both fish and amniotes, there is a switch during hindbrain development from an early pattern of Fgf signalling conferring segmental identity to a late regime of Fgf expression regulating neurogenesis in specific, sub-rhombomeric domains. Nonetheless, the differences in the ligands involved and their expression patterns point to species-specific features in the regulation of Fgf signalling in the hindbrain. In chicken, BMP signalling (Weisinger et al., 2008), as well as an unidentified signal from the boundaries (Sela-Donenfeld et al., 2009), have been implicated in the downregulation of *Fgf3* outside the boundaries; however, how the switch between two different patterns of Fgf signalling is coordinated is not understood.

The zinc finger and BTB domain containing 16 (Zbtb16) transcription factor – also known as promyelocytic leukaemia zinc finger (Plzf) – regulates a number of different processes during normal development. Zbtb16 opposes differentiation and maintains a progenitor state in haematopoietic and spermatogenetic stem cells (Shaknovich et al., 1998; Buaas et al., 2004; Costoya et al., 2004) but promotes differentiation of megakaryocytes (Labbaye et al., 2002), chondrogenesis (Liu et al., 2011) and osteogenesis (Agrawal Singh et al., 2019). Zbtb16 is expressed widely in the embryonic amniote CNS, including the hindbrain (Avantaggiato et al., 1995; Cook et al., 1995; Tailor et al., 2013), as well as in the early neural epithelium in zebrafish (Sobieszczuk et al., 2010). In addition, Zbtb16 is expressed specifically in rosette-forming human embryonic stem cell-derived neural stem cells, a stage exhibiting a wide differentiation potential (Elkabetz et al., 2008). In mouse and chicken embryos, *zbtb16* mRNA is initially enriched in even-numbered rhombomeres and is gradually confined to rhombomere boundaries as development proceeds (Cook et al., 1995). In zebrafish, Zbtb16 acts as an antagonist of neural differentiation during the development of primary neurons, and its degradation is required for neurogenesis to proceed (Sobieszczuk et al., 2010). In chicken and mouse, Zbtb16 maintains spinal cord neural progenitors in an undifferentiated state by positively regulating Fgf receptor expression (Gaber et al., 2013). These results imply a conserved role for Zbtb16 in neural progenitor maintenance.

Here, we examine the expression and function of Zbtb16 in the zebrafish embryonic hindbrain. We find that Zbtb16 has a dynamic pattern of expression and is initially enriched in specific rhombomeres, and that Zbtb16 expression is largely confined to neural progenitors and absent from postmitotic neurons. To assess the function of Zbtb16 in hindbrain development, we generated *zbtb16a*/*b* double mutants, which reveal a role for Zbtb16 in downregulating the early segment-specific expression of *fgf3*, required for the correct organization of neurogenesis at later stages. Our results point to a previously undescribed role for Zbtb16 in regulating Fgf ligand expression and show that Zbtb16 function is essential for coordinating the switch between early and late patterns of Fgf signalling in the zebrafish hindbrain.

## RESULTS

Previous work has implicated Zbtb16 in the regulation of neurogenesis in the zebrafish CNS, and provided evidence that it acts as an inhibitor of proneural gene expression (Sobieszczuk et al., 2010). This predicts that loss of function of Zbtb16 paralogues will lead to excessive and/or premature neurogenesis. To test this, we generated null mutations in the Zbtb16 paralogues, *zbtb16a* and *zbtb16b*. Unexpectedly, we found altered patterning and a reduced level of neurogenesis. Interpretation of these results required a more detailed understanding of the expression pattern of Zbtb16 paralogues and their relationship to neurogenesis.

### Dynamic expression of Zbtb16 in the zebrafish hindbrain

**Fig. 1.**
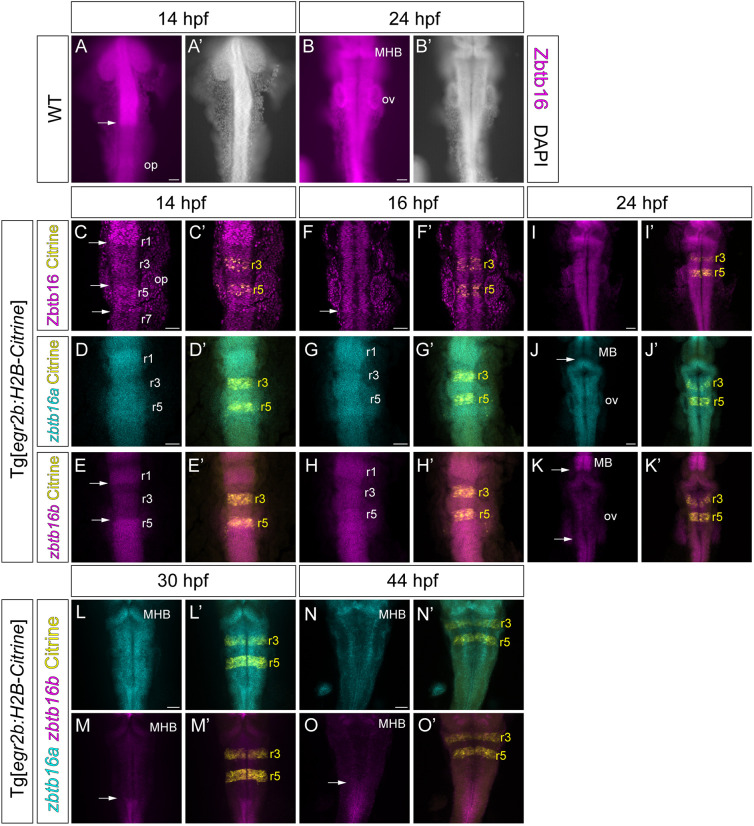
**Expression of Zbtb16 protein and *zbtb16a*/*b* mRNA in the zebrafish hindbrain.** (A,B) Wholemount fluorescence micrographs showing staining with anti-Zbtb16 antibody (see Materials and Methods) at 14 hpf and 24 hpf. (C,F,I) Immunofluorescent staining with anti-Zbtb16 antibody at 14, 16 and 24 hpf. C and F show slices from confocal *z*-stack, I shows sum projection through *z*-stack. n≥5 embryos per stage. (D,E,G,H,J-O) HCR for *zbtb16a* and *zbtb16b* at 14, 16, 24, 30 and 44 hpf. Sum projections through the *z*-stack. *n*≥5 embryos per stage. The Tg[*egr2b:H2B-Citrine*] line expresses Citrine in rhombomeres (r) 3 and 5. Arrows indicate boundaries between high and low levels of Zbtb16 protein or *zbtb16a*/*b* mRNA expression. MB, midbrain; MHB, midbrain-hindbrain boundary; op, otic placode; ov, otic vesicle. Scale bars: 50 µm.

Previous studies have shown that *zbtb16a* and *zbtb16b* are expressed in the zebrafish CNS, including a part of the hindbrain, at 10-12 h postfertilization (hpf) (Sobieszczuk et al., 2010), but have not examined their temporal and spatial pattern in detail, particularly at later stages. To address this, we used hybridization chain reaction (HCR; Choi et al., 2018) *in situ* hybridization (ISH) to detect *zbtb16a* and *zbtb16b*, and used a transgenic reporter line that expresses Citrine in r3 and r5 to ascertain the identity of individual rhombomeres (Addison et al., 2018). In addition, we carried out immunodetection with an antibody that detects both Zbtb16 paralogues (Fig. S1). High level Zbtb16 protein expression is confined to the anterior CNS including the anterior hindbrain at 14 hpf (Fig. 1A,A′), but by 24 hpf has expanded throughout the hindbrain and spinal cord (Fig. 1B,B′). To characterize this in more detail, we carried out HCR and immunodetection at 14 hpf, 16 hpf and 24 hpf (Fig. 1C-K′). At 14 hpf and 16 hpf, *zbtb16a* is expressed widely in the hindbrain, with somewhat lower levels in r7 (Fig. 1D,G). *zbtb16b* has a more rhombomere-specific expression pattern at 14 hpf, with higher levels of expression in r1 and r5-r7 as well as the anterior spinal cord (Fig. 1E). At 16 hpf, *zbtb16b* levels are higher in r2-r4, leading to a more uniform pattern of expression in the hindbrain (Fig. 1H). Immunodetection using the r3/r5 reporter line at 14 hpf reveals a sharp decrease in Zbtb16 protein levels posterior to r1 and elevated levels in r5-r6 (Fig. 1C). By 16 hpf, Zbtb16 protein levels are more uniform anterior of the r6/r7 boundary, whereas more caudal regions show relatively lower levels of expression (Fig. 1F). This pattern of expression may be accounted for by higher Zbtb16 protein levels in segments in which both *zbtb16a* and *zbtb16b* mRNAs are expressed.

At 24 hpf, *zbtb16a* is expressed widely across the hindbrain and the anterior spinal cord, whereas *zbtb16b* levels appear to be low in the hindbrain (Fig. 1J,K). In accordance with this, Zbtb16 protein is present at uniform levels across the whole hindbrain and anterior spinal cord (Fig. 1I). In addition, *zbtb16b* mRNA is enriched in the midbrain in a domain with low levels of *zbtb16a* expression (Fig. 1J,K). Together, these results reveal that Zbtb16 expression begins in the anterior hindbrain and by 24 hpf has expanded into the posterior hindbrain and anterior spinal cord.

**Fig. 2.**
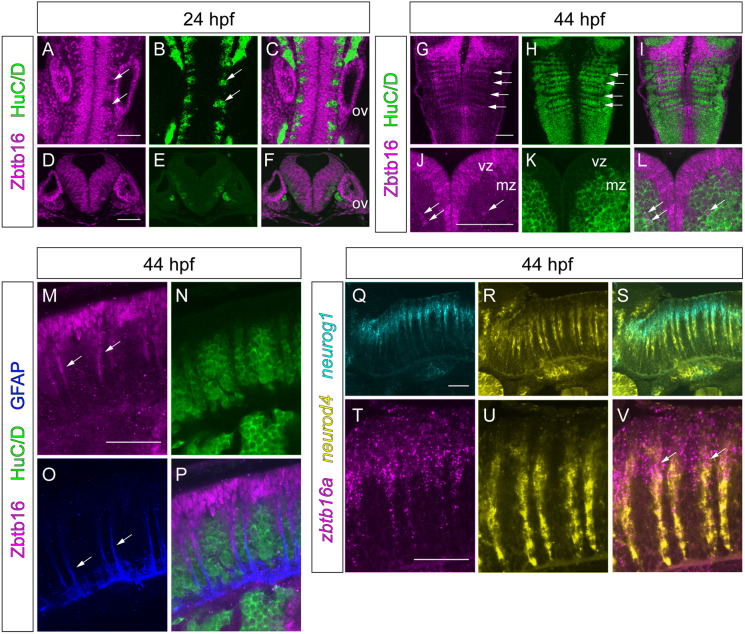
**Expression of Zbtb16 during neurogenesis.** (A-L) Immunofluorescence for Zbtb16 and HuC/D at 24 and 44 hpf. Dorsal views (A-C,G-I) and transverse sections (D-F,J-L) of the hindbrain. G-I are coronal slices at the level of the mantle zone. Arrows indicate postmitotic neurons (A,B), neurogenic zones (G,H) or Zbtb16+HuC/D double-positive cells (J,L). (M-P) Immunofluorescence for Zbtb16, HuC/D and GFAP at 44 hpf. Side views of the hindbrain, anterior to the left. Arrows indicate Zbtb16-positive migrating progenitors (M) or GFAP-positive glial fibres (O). (Q-V) Two-colour fluorescent ISH for *neurog1* and *neurod4* (Q-S) or *zbtb16a* and *neurod4* (T-V) at 44 hpf. Side views of the hindbrain, anterior to the left. Arrows indicate overlap of *zbtb16a* and *neurod4* expression. All images are slices from confocal *z*-stacks. mz, mantle zone; ov, otic vesicle; vz, ventricular zone. Scale bars: 50 µm.

At 24 hpf, neurogenesis in the hindbrain is being patterned into neurogenic zones adjacent to rhombomere boundaries, which have become fully established by 30 hpf. We carried out HCR to analyse Zbtb16 paralogue expression at 30 hpf and 44 hpf (Fig. 1L-O′). We found that *zbtb16a* has widespread expression at 30 hpf and is expressed at apparently lower levels throughout the hindbrain at 44 hpf; this may reflect a change in the relative size of the ventricular zone (see Fig. 2). *zbtb16b* has low expression in the hindbrain at both 30 hpf and 44 hpf, like the situation at 24 hpf.

### Expression of Zbtb16 in relation to neurogenesis

As Zbtb16 has been implicated in progenitor maintenance and repressing neuronal differentiation, we characterized its expression in relation to neurogenesis in more detail. We combined Zbtb16 immunostaining with co-staining for neuronal and neural progenitor markers at 24 and 44 hpf, stages which fall within the major phase of neurogenesis in the zebrafish hindbrain (Lyons et al., 2003). At 24 hpf, the ventricular zone, which contains the neural progenitors, occupies most of the hindbrain. Zbtb16 protein is present throughout the hindbrain ventricular zone but absent from the HuC/D-positive postmitotic neurons (Fig. 2A-F). At 44 hpf, the mantle zone containing postmitotic neurons has grown at the expense of the ventricular zone, and the latter has acquired a characteristic ‘T-shape’ (Lyons et al., 2003). Zbtb16 is expressed throughout the ventricular zone but is absent from the mantle zone, except for a few postmitotic neurons (Fig. 2J-L).

At the 44 hpf stage, neurogenesis in the hindbrain is localized to narrow stripes between the rhombomere centres and boundaries, the neurogenic zones (Gonzalez-Quevedo et al., 2010). The neurogenic zones contain radial glial fibres that extend from the ventricular zone to the pial surface of the mantle zone (Trevarrow et al., 1990). These fibres exclude neurons, thus creating gaps in HuC/D staining seen in coronal confocal sections through the mantle zone, i.e. below the plane of the ventricular zone (Fig. 2H). Cells in these gaps express Zbtb16 (Fig. 2G-I). Side views of the hindbrain show Zbtb16 staining in stripes of cells that extend ventrally from the ventricular zone and are negative for HuC/D staining (Fig. 2M,N). Co-staining for glial fibrillary acid protein (GFAP) reveals that the Zbtb16-expressing cell stripes coincide with glial fibres (Fig. 2M-P). Furthermore, most hindbrain neuronal progenitors at 44 hpf are radial glia (Lyons et al., 2003). Our observations therefore suggest that Zbtb16 is expressed in all neuronal progenitors in the ventricular zone and at early stages of migration along glial fibres from the neurogenic zone to the mantle zone as they differentiate into neurons.

To pinpoint the state of differentiation of Zbtb16-expressing cells further, we examined the expression of *zbtb16a* mRNA in relation to proneural gene expression. *neurog1* is expressed in undifferentiated progenitors and cells that have started to differentiate, whereas the intermediate proneural gene *neurod4* is expressed later in the neurogenic cascade in cells that have migrated out of the ventricular zone and occupy a more basal position (Fig. 2Q-S). *zbtb16a* mRNA is detected in the ventricular zone as well as in early migrating progenitors, where its expression is apical to that of *neurod4* with a region of overlap (Fig. 2T-V), indicating co-expression of *zbtb16a* and *neurog1*. Taken together, these results show that *zbtb16a* is expressed in both undifferentiated and early differentiating neuronal progenitors, downregulated upon migration out of the ventricular zone, and mostly absent from postmitotic neurons.

### The patterning of neurogenic zones is disrupted in Zbtb16 mutants

To assess the role of Zbtb16 in hindbrain neurogenesis, we took advantage of transcription activator-like effector nuclease (TALEN)-mediated targeted mutagenesis to generate zebrafish mutants for each of the Zbtb16 paralogues. The mutations are predicted to generate null alleles and, indeed, no Zbtb16 protein could be detected in double homozygous embryos after staining with Zbtb16 antibody (Fig. S2C-F). Furthermore, immunostaining of *zbtb16a* homozygous mutants found no detectable Zbtb16 protein at 24 hpf, and knockdown of *zbtb16b* in *zbtb16a* heterozygous mutants did not lead to any reduction in Zbtb16 protein levels. These data suggest that from 24 hpf onwards, only *zbtb16a* contributes to Zbtb16 protein production (Fig. S2G-L).

**Fig. 3.**
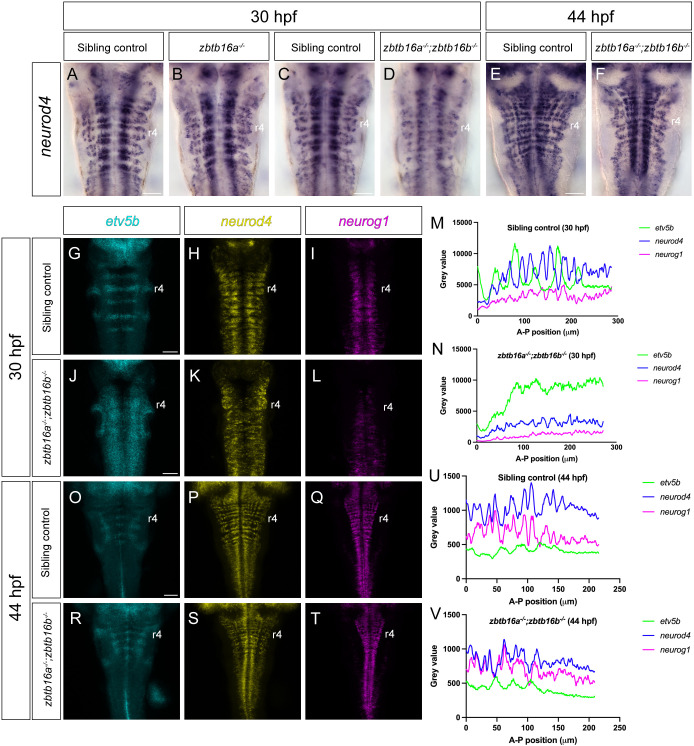
**Spatial pattern of neurogenesis in Zbtb16 mutants.** (A-F) Colorimetric ISH for *neurod4* in 30 hpf sibling control (*n*=121) and *zbtb16a^−/−^* (*n*=7) embryos (A,B); 30 hpf sibling control (*n*=18) and *zbtb16a^−/−^*;*zbtb16b^−/−^* (*n*=8) embryos (C,D); 44 hpf sibling control (*n*=8) and *zbtb16a^−/−^*;*zbtb16b^−/−^* (*n*=8) embryos (E,F). (G-L,O-T) HCR for *etv5b*, *neurod4* and *neurog1* in 30 hpf sibling control (*n*=16) and *zbtb16a^−/−^*;*zbtb16b^−/−^* (*n*=11) embryos (G-L); 44 hpf sibling control (*n*=9) and *zbtb16a^−/−^*;*zbtb16b^−/−^* (*n*=12) embryos (O-T). Sum projections through confocal *z*-stack. (M,N,U,V) Profile plots of *etv5b*, *neurod4* and *neurog1* HCR signal across the hindbrain of representative sibling control (*zbtb16a^+/+^*;*zbtb16b^−/−^*) and *zbtb16a^−/−^*;*zbtb16b^−/−^* embryos; anterior to the left. Scale bars: 50 µm.

As *zbtb16a* but not *zbtb16b* is expressed at late stages in the hindbrain, we analysed *zbtb16a* null mutants as well as *zbtb16a^−/−^*;*zbtb16b^−/−^* double mutants at 30 hpf and 44 hpf. At these stages, the neurogenic zones of the hindbrain are well delineated and can be visualized by detection of *neurod4* mRNA. At 30 hpf, we found no change in the organization of neurogenesis in *zbtb16a* single mutant embryos (Fig. 3A,B), but neurogenesis appeared to be decreased and disorganized in *zbtb16a^−/−^*;*zbtb16b^−/−^* double mutant embryos (Fig. 3C,D). However, by 44 hpf the pattern of neurogenesis revealed by *neurod4* expression was similar in wild-type and double mutant embryos (Fig. 3E,F). Thus, rather than leading to increased neurogenesis as predicted from previous studies, loss of Zbtb16 function appeared to lead to a transient decrease and disorganization of neurogenesis.

Previous studies have found that neurogenesis is organized at these stages by *fgf20*-expressing neurons located at rhombomere centres that locally inhibit neurogenesis, thus confining it to zones adjacent to boundaries (Gonzalez-Quevedo et al., 2010). Consequently, expression of the ETS transcription factor Etv5b, a target of Fgf signalling (Münchberg et al., 1999; Raible and Brand, 2001; Roehl and Nüsslein-Volhard, 2001) occurs at rhombomere centres (Gonzalez-Quevedo et al., 2010). We therefore analyzed the pattern of neurogenesis and Fgf signalling in Zbtb16 double mutant and sibling control (*zbtb16b* homozygous mutant) embryos by HCR of *neurog1*, *neurod4* and *etv5b* followed by quantification (Fig. 3G-V). We found that at 30 hpf, expression of *etv5b* is widespread in the hindbrain in double mutant embryos, in contrast to the stripes at rhombomere centres in controls (Fig. 3G,J). The expression of *neurog1* and *neurod4* is at lower levels and disorganized in the double mutant embryos compared with controls (Fig. 3H,I,K,L), This is confirmed by the HCR signal profiles, which show the complementarity of *etv5b* and *neurod4* gene expression in sibling controls; *neurog1* expression is less strongly patterned as it also occurs in progenitors (Fig. 3M). In double mutants there is more uniform *etv5b*, *neurog1* and *neurod4* expression, and the neurogenic genes are expressed at lower levels (Fig. 3N; Fig. S3). Analysis of 44 hpf embryos revealed that by this stage the pattern and levels of *etv5*, *neurog1* and *neurod4* in double mutant embryos are very similar to wild type, although subtle patterning defects may persist in ventral progenitors marked by *neurog1* (Fig. 3O-V).

These results reveal that the spatial pattern of neurogenesis is transiently disrupted in embryos lacking Zbtb16. As Fgf signalling inhibits neurogenesis in rhombomere centres (Gonzalez-Quevedo et al., 2010), the altered pattern of neurogenesis upon loss of Zbtb16 may be a consequence of excess and more widespread Fgf expression. Furthermore, as the patterning defect is only manifest upon loss of both Zbtb16 paralogues, the phenotype likely stems from a stage before 24 hpf, when both paralogues are expressed in the hindbrain.

### Loss of Zbtb16 leads to excess Fgf signalling in the hindbrain at 24 hpf

From approximately 20 hpf, Fgf20 signalling from clusters of early-born neurons inhibits neurogenesis in rhombomere centres, resulting in neurogenic and non-neurogenic zones in the hindbrain (Gonzalez-Quevedo et al., 2010). Our finding that the Fgf target *etv5b* is ectopically expressed in the hindbrain at 30 hpf in embryos lacking Zbtb16 prompted us to examine the relationship between Zbtb16 and Fgf signalling at an earlier stage.

**Fig. 4.**
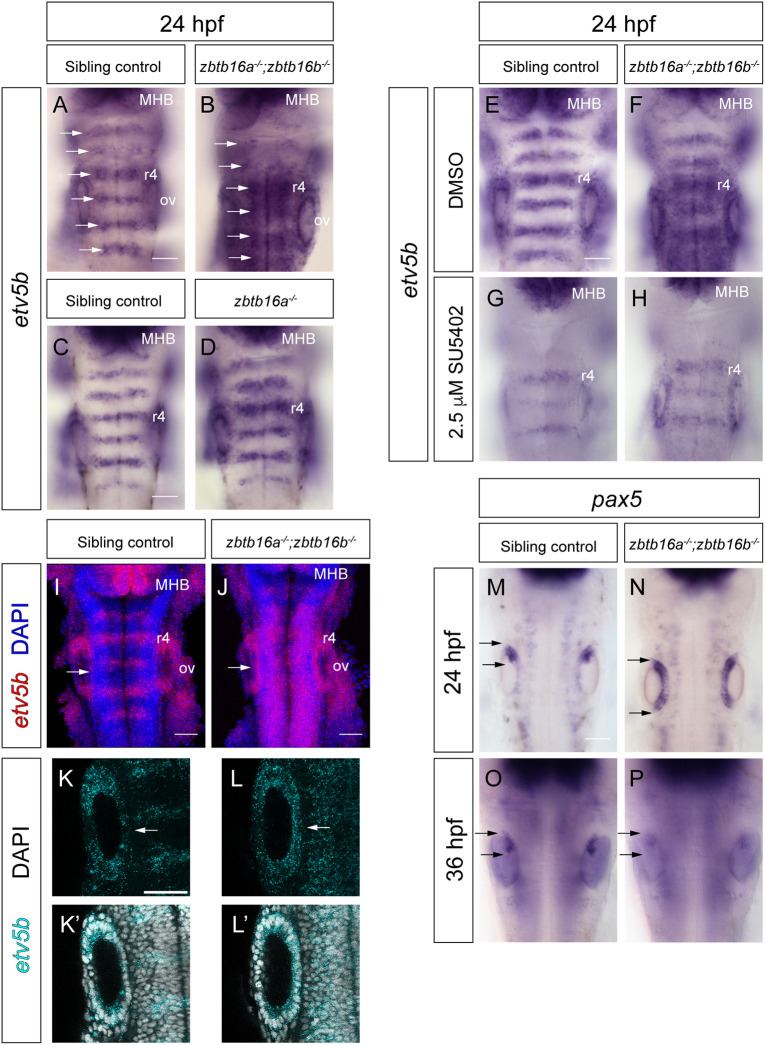
**Spatial analysis of Fgf signalling in Zbtb16 mutants.** (A,B) Colorimetric ISH for *etv5b* in sibling control (A; *n*=59) and *zbtb16a^−/−^*;*zbtb16b^−/−^* (B; *n*=25) embryos. Dorsal views of the hindbrain, wholemount. Arrows indicate rhombomere centres. (C,D) ISH for *etv5b* in sibling control (C; *n*=18) and *zbtb16a^−/−^* (D; *n*=6) embryos. (E,F) Sibling control (E; *n*=88) and *zbtb16a^−/−^*;*zbtb16b^−/−^* (F; *n*=28) embryos treated with DMSO from 22 to 24 hpf and stained for *etv5b*. (G,H) Sibling control (G; *n*=73) and *zbtb16a^−/−^*;*zbtb16b^−/−^* (H; *n*=38) embryos treated with 2.5 µM SU5402 from 22 to 24 hpf and stained for *etv5b*. Three independent experiments. (I-L′) HCR for *etv5b* in 24 hpf; sibling control (*n*=14) and *zbtb16a^−/−^*;*zbtb16b^−/−^* (*n*=9) mutant embryos. 3D reconstructions of the *z*-stack, dorsal view (I,J); confocal *z*-stack slices (K-L). Arrows in I-L′ indicate the medial wall of the otic vesicle. (M,N) Colorimetric ISH for *pax5* in 24 hpf sibling control (*n*=10) and *zbtb16a^−/−^*;*zbtb16b^−/−^* (*n*=11) embryos. (O,P) ISH for *pax5* in 36 hpf sibling control (*n*=29) and *zbtb16a^−/−^*;*zbtb16b^−/−^* (*n*=11) embryos. Arrows in M-P show the antero-posterior extent of marker gene expression in the otic vesicle. MHB, midbrain-hindbrain boundary; ov, otic vesicle; r, rhombomere. Scale bars: 50 µm.

We analysed *etv5b* expression in zebrafish embryos with mutations in *zbtb16a* alone (*zbtb16a^−/−^*) and both *zbtb16a* and *zbtb16b* (*zbtb16a^−/−^*;*zbtb16b^−/−^*). In sibling controls (*zbtb16b* homozygous mutant), *etv5b* mRNA is confined to rhombomere centres at 24 hpf, as occurs in wild-type embryos. By contrast, in *zbtb16a^−/−^*;*zbtb16b^−/−^* mutant embryos, *etv5b* expression is detected outside rhombomere centres (Fig. 4A,B,I,J). Ectopic *etv5b* expression is also observed posterior to r7 in the caudal hindbrain. By contrast, the *zbtb16a^−/−^* single mutant shows a comparatively mild phenotype, with some ectopic *etv5b* typically only detected in r4 (Fig. 4C,D).

To determine whether the ectopic expression *of etv5b* in the hindbrain observed after Zbtb16 loss of function is a consequence of excess Fgf signalling, we inhibited Fgf receptor signalling in *zbtb16a^−/−^*;*zbtb16b^−/−^* embryos with SU5402 (Mohammadi et al., 1997). Treating embryos from 22 to 24 hpf with 2.5 µM SU5402 was sufficient to abolish *etv5b* expression partially or completely in the hindbrain of both sibling controls and *zbtb16a^−/−^*;*zbtb16b^−/−^* embryos, compared with vehicle controls (Fig. 4E-H). Together, these results demonstrate that loss of both Zbtb16 paralogues leads to excess Fgf signalling in the hindbrain at 24 hpf, suggesting a role for Zbtb16 in negatively regulating Fgf signalling or expression.

In addition, we observe ectopic *etv5b* expression in the medial wall of the otic vesicle at 24 hpf (Fig. 4I-L). In zebrafish, Fgf signalling from r4 acts as an anteriorizing signal to pattern the otic vesicle (Kwak et al., 2002; Hammond and Whitfield, 2011; Hartwell et al., 2019). We therefore assayed Zbtb16 double mutants for expression of the anterior markers *pax5* and *hmx2*, which expand posteriorly in response to Fgf misexpression (Kwak et al., 2002; Hammond and Whitfield, 2011). Indeed, we found that *pax5* and *hmx2* expression is detected throughout the medial wall of the otic vesicle at 24 hpf (Fig. 4M,N; Fig. S4A,B). However, full mirror image duplication of the ear (Hartwell et al., 2019) is not observed, as *pax5* is confined to the anterior macula at 36 hpf in both controls and double mutants (Fig. 4O,P). In contrast, ectopic expression of *pax5* is never detected in the *zbtb16a^−/−^* otic vesicle (Fig. S4C,D). As *zbtb16a*, but not *zbtb16b*, is expressed in the otic vesicle (Fig. 1; Fig. S4E,F), this result argues against a tissue-autonomous phenotype in the inner ear, and instead suggests that the expansion of anterior markers is due to increased Fgf signalling from the hindbrain. Together, these results suggest that the inner ear is transiently anteriorized in the absence of Zbtb16, likely as a consequence of ectopic Fgf signalling from the hindbrain.

### The expression of *fgf3* persists in the Zbtb16 mutant hindbrain

**Fig. 5.**
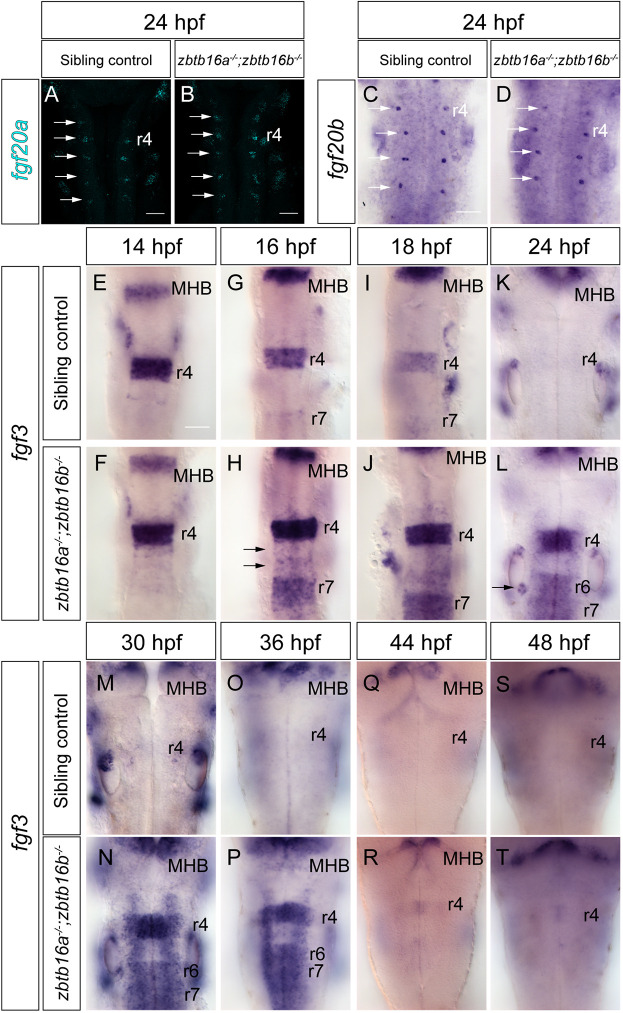
**Expression of Fgf ligands in Zbtb16 mutants.** (A,B) HCR for *fgf20a* in 24 hpf sibling control (*n*=26) and *zbtb16a^−/−^*;*zbtb16b^−/−^* (*n*=12) embryos. 3D reconstructions of the *z*-stack, dorsal view. (C,D) Colorimetric ISH for *fgf20b* in 24 hpf sibling control (*n*=15) and *zbtb16a^−/−^*;*zbtb16b^−/−^* (*n*=4) embryos. Arrows in A-D indicate *fgf20-*expressing neuronal clusters in rhombomere centres. (E-T) ISH time-course of *fgf3* expression in the sibling control (14 hpf: *n*=19; 16 hpf: *n*=26; 18 hpf: *n*=53; 24 hpf: *n*=47; 30 hpf: *n*=10; 36 hpf: *n*=35; 44hpf: *n*=20; 48 hpf: *n*=43) and *zbtb16a^−/−^*;*zbtb16b^−/−^* (14 hpf: *n*=6; 16 hpf: *n*=10; 18 hpf: *n*=20; 24 hpf: *n*=12; 30 hpf: *n*=5; 36 hpf: *n*=13; 44 hpf: *n*=10; 48 hpf: *n*=12) hindbrain. Arrows in H indicate ectopic expression in r5/r6, arrow in L indicates ectopic expression in the posterior otic vesicle. MHB, midbrain-hindbrain boundary; r, rhombomere. Scale bars: 50 µm.

A possible explanation for ectopic Fgf signalling in the Zbtb16 double mutants is the ectopic expression of one or more Fgf ligands in the hindbrain. In the wild type, at 24 hpf, *fgf20a* is expressed in clusters of early-born neurons located bilaterally in the centre of each rhombomere and accounts for the majority of *etv5b* expression in rhombomere centres (Gonzalez-Quevedo et al., 2010). Staining for *fgf20a* did not reveal any difference in expression between *zbtb16a^−/−^*;*zbtb16b^−/−^* and control embryos (Fig. 5A,B). The paralogous gene *fgf20b* is typically expressed in one or a few neurons within each *fgf20a*-expressing cluster, and this is also the case in *zbtb16a^−/−^*;*zbtb16b^−/−^* mutants (Fig. 5C,D). Therefore, a broader source of Fgf20 signalling does not account for the phenotype observed in embryos lacking Zbtb16.

An alternative source of ectopic Fgf signalling could be a ligand which is normally transiently expressed at an earlier stage of development and which persists in the mutant. In zebrafish, *fgf8* and *fgf3* are expressed in the prospective hindbrain from late gastrulation in a domain that becomes restricted to r4. These Fgf family members are required for correct segmental gene expression in the adjacent rhombomeres, as well as induction of the inner ear (Phillips et al., 2001; Maroon et al., 2002; Maves et al., 2002; Walshe et al., 2002). *fgf8* is downregulated in r4 soon after hindbrain segmentation (Walshe et al., 2002), whereas *fgf3* expression persists until later and is downregulated by ∼19 hpf (Maroon et al., 2002; Reuter et al., 2019).

To determine whether *fgf3* or *fgf8* expression persists in the hindbrain, we examined their expression in *zbtb16a^−/−^*;*zbtb16b^−/−^* mutant embryos and sibling controls. At 14 hpf, *fgf3* is expressed in r4 at similar levels in mutants and controls (Fig. 5E,F), whereas at 16 hpf double mutant embryos show high levels of *fgf3* in r4 compared with controls, and ectopic *fgf3* expression is observed in r5 and r6 (Fig. 5G,H). In sibling controls, low levels of *fgf3* are consistently detected caudally of the r6/7 boundary at 16 hpf, whereas expression levels in these domains are higher in the double mutants (Fig. 5G,H). At 18 hpf, ectopic *fgf3* persists in r5 and r6 and *fgf3* levels remain high in r7 and more caudally (Fig. 5I,J). At this stage, the sibling controls are downregulating *fgf3* in r4, whereas high levels persist in r4 in the double mutants (Fig. 5I,J). By 24 hpf, no *fgf3* expression can be detected in the hindbrain of sibling controls, but expression persists in Zbtb16 double mutants in r4, at intermediate levels in r6, r7 and more caudally, and at low levels in r5 (Fig. 5K,L). This expression of *fgf3* in r4, r6 and r7 persists at 30 hpf and 36 hpf (Fig. 5M-P); some expression is also detected in r3 and r5 in a dorsal domain. By 44 hpf, low levels of *fgf3* expression are detected in the hindbrain (Fig. 5Q,R) and, at 48 hpf, only residual expression is present (Fig. 5S,T).

*fgf3* expression in the hindbrain caudal to r6 has not been described previously in wild-type embryos; as the sibling controls lack functional *zbtb16b*, the low-level expression of *fgf3* detected at these axial levels could be ectopic expression caused by *zbtb16b* loss of function. We therefore examined *fgf3* expression in wild-type embryos at 16 hpf and found that low levels are present posterior to the r6/7 boundary (Fig. S5). This suggests that the ectopic *fgf3* detected in these tissues in Zbtb16 double mutants at 24 hpf represents a failure to downregulate *fgf3* in its normal expression domain. By contrast, *fgf8* expression is not detected in the hindbrain of sibling controls or double mutants at any of the stages examined (Fig. S6A-H).

Taken together, these results indicate that Zbtb16 downregulates *fgf3* expression in the hindbrain between 14 hpf and 24 hpf. This timing is consistent with the expression pattern of Zbtb16, with the onset of higher levels of Zbtb16 protein in the posterior hindbrain (Fig. 1F,G,H) preceding the downregulation of *fgf3* (Fig. 5E-H). One possible explanation is that changes in *fgf3* expression are secondary to altered expression of segment identity genes such as *hoxb1a*, which is expressed in r4. However, we detected no change in *hoxb1a* or *hoxb2* expression in Zbtb16 double mutants (Fig. S6I-L). Furthermore, the selective change in *fgf3* but not *fgf8* expression in Zbtb16 mutants is not consistent with global changes in segment identity. Although we cannot exclude the contribution of other Fgf ligands, our results suggest that altered *fgf3* expression is responsible for the persistent and ectopic Fgf signalling observed in the absence of Zbtb16.

### Fgf3 knockdown partially rescues Zbtb16 loss of function

**Fig. 6.**
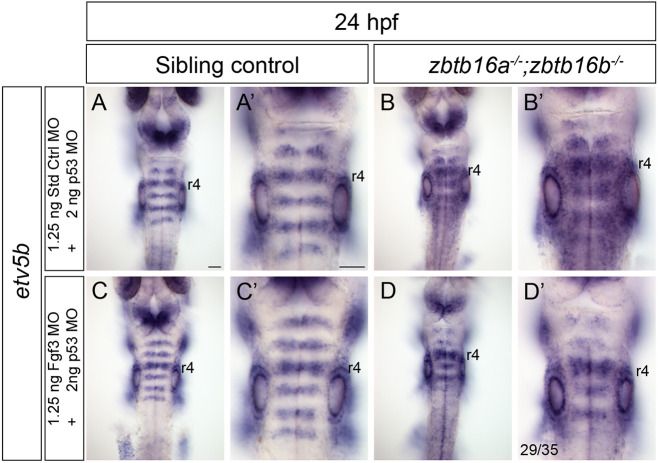
**Partial rescue of the Zbtb16 mutant phenotype by Fgf3 knockdown.** (A-B′) Colorimetric ISH for *etv5b* in sibling control (*n*=88) and *zbtb16a^−/−^*;*zbtb16b^−/−^* (*n*=40) embryos injected with standard control+*p53* MO. (C-D′) ISH for *etv5b* in sibling control (*n*=83) and *zbtb16a^−/−^*;*zbtb16b^−/−^* (*n*=35) embryos injected with Fgf3 translation blocking MO+*p53* MO. Three independent experiments. Dorsal views of the hindbrain, wholemount. r, rhombomere. Scale bars: 50 µm.

Our proposal that continued segmental expression of *fgf3* expression at late stages in Zbtb16 mutants underlies ectopic Fgf signalling predicts that the latter will be rescued by knockdown of *fgf3*. We therefore performed morpholino (MO)-mediated knockdown of *fgf3* in embryos derived from incrosses of *zbtb16a^+/−^*;*zbtb16b^−/−^* adults and carried out genotyping to identify double mutant embryos; *p53* MO is co-injected as this suppresses side-effects of MO toxicity (Robu et al., 2007; Gerety and Wilkinson, 2011) (Fig. 6A-D′). Of the *zbtb16a^−/−^*;*zbtb16b^−/−^* embryos injected with an *fgf3* translation-blocking MO, 29/35 (83%) exhibited a partial rescue of the pattern of Fgf signalling at 24 hpf, with *etv5b* expression occurring in stripes in the hindbrain (Fig. 6D), albeit broader than in sibling controls (Fig. 6C); in 2/35 (6%) the phenotype was completely rescued, and 4/35 (11%) showed no rescue. The domain of *etv5b* was always broadest in r4 in partially rescued embryos, consistent with the levels of persistent *fgf3* being highest in that segment (Fig. 5). By contrast, expression of *etv5b* occurs throughout the hindbrain in double homozygous embryos injected with a control MO (40/40) (Fig. 6B). All sibling controls injected with either the *fgf3* MO (83/83) or control MO (88/88) showed normal stripes of *etv5b* expression, suggesting that *fgf3* knockdown does not interfere with the later pattern of Fgf signalling in rhombomere centres (Fig. 6A,C), which is mediated by *fgf20* (Gonzalez-Quevedo et al., 2010). The range of rescued phenotypes obtained by Fgf3 depletion in *zbtb16a^−/−^*;*zbtb16b^−/−^* embryos strongly suggests that the persistence of *fgf3* expression is the source of the ectopic Fgf signalling observed upon loss of Zbtb16.

### Overexpression of Zbtb16 inhibits *fgf3* expression

**Fig. 7.**
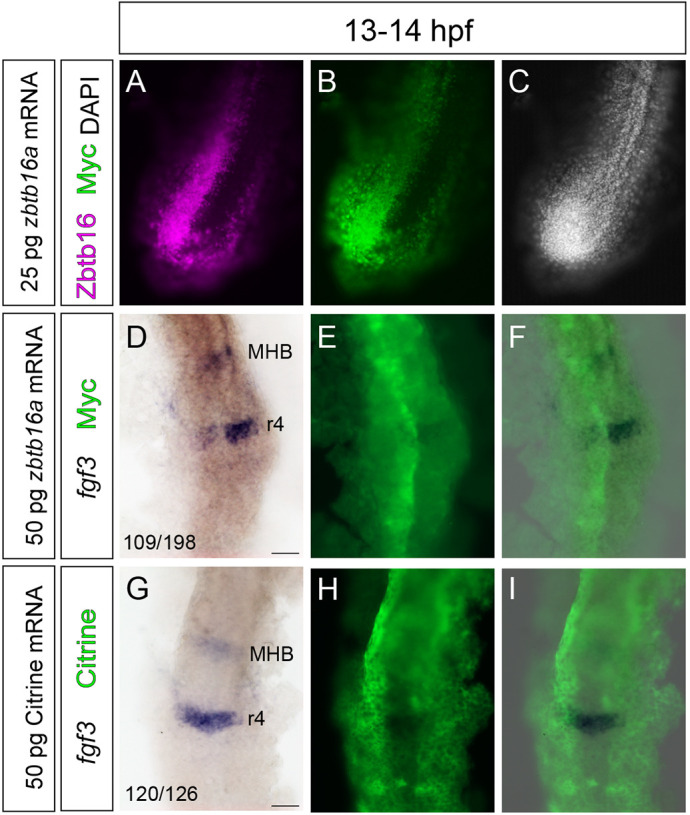
**Early misexpression of Zbtb16 downregulates *fgf3* in rhombomere 4.** (A-C) Wholemount fluorescence micrographs of the tailbud of a wild-type embryo injected in one half with mRNA encoding myc-Zbtb16a and stained with anti-Zbtb16 and anti-Myc antibodies. No endogenous Zbtb16 staining is detected at this axial level at 13-14 hpf. (D-F) ISH for *fgf3* followed by anti-Myc immunofluorescent staining in wild-type embryos (*n*=198) injected in one half with mRNA encoding myc-Zbtb16a. (G-I) ISH for *fgf3* in wild-type embryos (*n*=126) injected in one half with mRNA encoding Citrine, showing Citrine fluorescence. Three independent experiments. MHB, midbrain-hindbrain boundary; r, rhombomere. Numbers (D,G; bottom left) indicate numbers of embryos that show dissimilar (D) or similar (G) levels of *fgf3* expression in the left and right halves of r4. Scale bars: 50 µm.

If Zbtb16 indeed downregulates segmental *fgf3* expression, overexpression of Zbtb16 should lead to premature downregulation. To test this, we micro-injected mRNA encoding Myc-tagged Zbtb16a into one blastomere at the two-cell stage, so that ectopic expression occurred in one half of the embryo. We confirmed that immunodetection of the Myc tag co-localized with ectopic Zbtb16 protein (Fig. 7A-C). *fgf3* expression in r4 at 13-14 hpf was analysed by *in situ* hybridization (ISH). We found that 109/198 (55%) of embryos injected with 50 pg *zbtb16a* mRNA showed decreased expression of *fgf3* unilaterally in r4, whereas only 6/126 (5%) of control embryos injected with 50 pg of Citrine mRNA showed weaker *fgf3* expression on one side (Fig. 7D,G). In addition, Zbtb16a overexpression typically led to neural tube malformations (Fig. 7D). Immunodetection of Myc after ISH of *zbtb16a*-injected embryos revealed that the area in which *fgf3* is downregulated is coincident with Myc-positive cells, with the highest fluorescence intensity typically corresponding to the most complete downregulation (Fig. 7D-F). *fgf3* downregulation also occurs in Myc-positive cells at the midbrain-hindbrain boundary. In contrast, Citrine fluorescence in control embryos frequently overlaps with high levels of *fgf3* staining (Fig. 7G-I). These data show that a premature increase in Zbtb16 levels downregulates *fgf3* expression in r4 and support a model in which rising levels of Zbtb16 shut down *fgf3* expression in the hindbrain between 14 hpf and 24 hpf.

## DISCUSSION

Embryonic tissues are sequentially patterned by signals which have stage-specific roles and may act through shared downstream pathways. Thus, where and when the expression of signalling pathway components is turned on and off must be tightly controlled during development. Here, we show that the transcription factor Zbtb16 is required for switching between stage-specific patterns of Fgf signalling in the zebrafish hindbrain. Zbtb16 protein expression is at low levels in the posterior hindbrain at 14 hpf, where *fgf3* expression occurs in r4, and is upregulated in this region during the period when segmental *fgf3* expression is downregulated. This downregulation is normally complete by 24 hpf but, in the absence of Zbtb16, *fgf3* expression persists up to 36 hpf, overlapping with the period when neurogenesis is patterned by localized Fgf20 signalling. The prolonged widespread pattern of Fgf signalling interferes with the organization of neurogenesis and transiently disrupts antero-posterior patterning of the inner ear.

### Zbtb16 acts at early stages to regulate Fgf signalling

The two Zbtb16 paralogues, *zbtb16a* and *zbtb16b*, have distinct temporal and spatial expression in the hindbrain, in which both are expressed at early stages, but only *zbtb16a* is expressed from 24 hpf. Consistent with this, in *zbtb16a* homozygous mutants, Zbtb16 protein is not detected in the hindbrain from 24 hpf onwards. However, the disruption to the patterning of Fgf signalling and neurogenesis occurs in *zbtb16a*; *zbtb16b* double mutants but not in *zbtb16a* single mutants. These findings suggest that the Zbtb16 paralogues act before 24 hpf, consistent with a role in the downregulation of segmental *fgf3* expression, which in turn is required subsequently for correct patterning of neurogenesis by fgf20.

As Zbtb16 can act as a transcriptional repressor, it is possible that it acts directly on the *fgf3* gene to counteract positive regulators. Alternatively, Zbtb16 may regulate other genes controlling *fgf3* expression. The homeobox transcription factor *hoxb1* is a known regulator of r4 identity (Studer et al., 1996; McClintock et al., 2002) and positively regulates *fgf3* expression in r4 (Waskiewicz et al., 2002; Choe et al., 2011; Weicksel et al., 2014). The fact that *hoxb1a* expression persists at high levels until at least 24 hpf in zebrafish, and the lack of ectopic *hoxb1a* expression in the *zbtb16a^−/−^*;*zbtb16b^−/−^* mutants, suggests that Zbtb16 is not regulating *fgf3* through *hoxb1.* GATA2 and GATA3 specify r4 identity downstream of Hoxb1 in the mouse hindbrain (Pata et al., 1999), and GATA4 mediates *fgf3* upregulation upon retinoic acid (RA)-induced differentiation of F9 embryonal carcinoma cells into parietal endoderm (Murakami et al., 1999), a cell type which also expresses *hoxb1* (Boylan et al., 1993). In a screen for downstream effectors of zebrafish *hoxb1b*, the protein phosphatase 1 regulatory subunit *ppp1r14al*, a potential regulator of GATA activity, was found to positively regulate *fgf3* expression in r4 (Choe et al., 2011). Intriguingly, Zbtb16 binds the mouse GATA4 regulatory region and upregulates its expression in heart cells (Wang et al., 2012b). GATA factors are thus candidate Zbtb16 targets in r4. Defining genome-wide Zbtb16 binding in neural tissue will shed light on the mechanism of *fgf3* downregulation by Zbtb16.

### Regulation of Zbtb16 expression

The sequential onset of Zbtb16 expression, first in the anterior CNS and then more posteriorly, suggests that regulation of Zbtb16 levels is coupled to an antero-posterior patterning process. During early hindbrain development, RA acts as a posteriorizing signal and forms a gradient across the hindbrain (reviewed by Schilling et al., 2016; Frank and Sela-Donenfeld, 2019). The hindbrain RA gradient has been proposed to be generated by rhombomere-specific expression of RA-metabolizing Cyp26 enzymes (Sirbu et al., 2005; Hernandez et al., 2007; White et al., 2007). In one model, the dynamic expression of different Cyp26 genes in the zebrafish hindbrain leads to a progressive posterior shift of the anterior border of the RA-responsive domain during somitogenesis stages (Hernandez et al., 2007). This suggests a model where low levels of RA signalling are permissive to Zbtb16 expression, which is upregulated in the posterior hindbrain as the domain of active RA signalling recedes. Other signalling pathways that regulate hindbrain antero-posterior patterning, such as Fgf and Wnt, as well as downstream transcription factor networks (reviewed by Parker and Krumlauf, 2017; Frank and Sela-Donenfeld, 2019) may also act upstream of Zbtb16. It will be interesting to ascertain whether reciprocal regulation occurs between Fgf signalling and Zbtb16 expression, and whether auto- or cross-regulation occurs between Zbtb16 paralogues.

### Zbtb16 and the Fgf signalling switch in fish and amniotes

The expression pattern of Zbtb16 in the zebrafish hindbrain differs from that of amniotes. Both zebrafish and chicken have different segmental levels of Zbtb16 expression during early hindbrain development, albeit in different patterns (Cook et al., 1995). At later stages, *Zbtb16* is expressed in rhombomere boundaries in chick, mouse and rat (Cook et al., 1995; Takahashi and Osumi, 2011). In chick embryos, the boundary cells constitute populations of slowly dividing, multipotent neuronal progenitors (Peretz et al., 2016). In contrast, in zebrafish, Zbtb16 is expressed throughout the ventricular region, including both the neurogenic zones and non-neurogenic rhombomere centres.

We found that *fgf3* expression in r4 in the early zebrafish hindbrain occurs when the level of Zbtb16 expression is low in this region, and *fgf3* expression persists in the absence of Zbtb16. In contrast, in amniotes, the expression pattern of Zbtb16 correlates with *fgf3* both in rhombomeres and later in rhombomere boundaries (Mahmood et al., 1995; Weisinger et al., 2008, 2010, 2012; Sela-Donenfeld et al., 2009). This suggests that the repression of *fgf3* by Zbtb16 is not a feature conserved between amniotes and fish. Thus, amniotes appear to have boundary-specific mechanisms for maintaining *fgf3* transcription, whereas zebrafish downregulate *fgf3* throughout the hindbrain by a mechanism involving Zbtb16.

During hindbrain development, a switch from an early pattern of Fgf signalling regulating rhombomere-specific properties to a later pattern regulating neurogenesis takes place. The early phase involves Fgf3 in both amniotes and fish (Weisinger et al., 2008, 2010; Maves et al., 2002; Walshe et al., 2002), whereas different Fgf ligands are used at the late phase: Fgf3 in amniotes (Weisinger et al., 2012) and Fgf20 in fish (Gonzalez-Quevedo et al., 2010). The use of different ligands may explain the distinct mechanisms for *fgf3* downregulation in the two groups. Fgf3 seems to act as a long-range signal as it is required for patterning of rhombomeres adjacent to r4. In contrast, homodimerization of Fgf20 and other ligands of the Fgf9 family increases their affinity to heparin, limiting their diffusivity (Kalinina et al., 2009; Harada et al., 2009). The short-range activity of Fgf20 may therefore be necessary to achieve the precise patterning of neurogenic zones within rhombomeres that occurs in zebrafish but not in amniotes.

### The role of Zbtb16 in neural progenitors

In human embryonic stem cell-derived neural stem cells, as well as those expanded from human embryonic hindbrain, Zbtb16 expression marks stages with a high capacity for progenitor maintenance and wide differentiation potential (Elkabetz et al., 2008; Tailor et al., 2013). Zbtb16-deficient mice have a smaller cerebral cortex with a reduced number of neurons, which may reflect a depletion of the neural stem cell pool (Lin et al., 2019). Consistent with these findings, Zbtb16 is expressed in the neural progenitors of the zebrafish hindbrain.

In addition to the ventricular zone, Zbtb16 expression occurs in early differentiating progenitors migrating out of the ventricular zone. This is consistent with the model of Sobieszczuk et al. (2010), in which the progression of neuronal differentiation requires that Zbtb16 is downregulated in progenitors selected to differentiate in the Notch-mediated lateral inhibition process. We found that Zbtb16 protein and *zbtb16a* mRNA expression overlap with that of *neurog1* but are largely mutually exclusive with *neurod4*. At the onset of neuronal differentiation, the Zbtb16 protein is targeted for degradation by the ubiquitin ligase adaptor protein Btbd6 (Sobieszczuk et al., 2010).

Zbtb16 has been implicated in the maintenance of neural progenitors in the CNS through negative regulation of neurogenesis (Sobieszczuk et al., 2010; Gaber et al., 2013). One potential mechanism by which Zbtb16 may antagonize neuronal differentiation is repression of proneural gene expression (Sobieszczuk et al., 2010), although this was not shown directly. In contrast, Gaber et al. (2013) demonstrate that Zbtb16 opposes differentiation of neural progenitors in the chicken and mouse spinal cord by upregulating Fgf receptor 3 (FGFR3). This upregulation increases Fgf signalling and alters the balance between cell proliferation and neurogenesis. Our results indicate that Zbtb16 negatively regulates Fgf signalling in the hindbrain by repressing *fgf3* expression. In accordance with the results of Gonzalez-Quevedo et al. (2010), we find that high levels of Fgf signalling correlate with reduced neurogenesis in *zbtb16a^−/−^;zbtb16b^−/−^* mutant fish. Thus, in both fish and amniotes, Zbtb16 acts to regulate neuronal differentiation by regulating components of the Fgf signalling pathway – in the former case by downregulating the expression of an Fgf ligand, and in the latter by positively regulating Fgf receptor expression levels.

The apparently paradoxical result that, in zebrafish, the loss of function of an antagonist of neurogenesis results in reduced neuronal differentiation may be explained by the earlier, stage-specific role of Zbtb16 in downregulating an Fgf signal. This excess Fgf signalling could mask loss of cell-intrinsic inhibition of neurogenesis by Zbtb16 in neural progenitors. However, we found no defect in neurogenesis resulting from loss of *zbtb16a* despite this paralogue contributing all Zbtb16 protein at 24 hpf and later; this may be interpreted in light of the finding that the effect of *zbtb16a* knockdown on primary neurogenesis only becomes manifest upon Notch inhibition (Sobieszczuk et al., 2010). Although it seems likely that the persistent expression of *fgf3* underlies the disruption in the pattern of neurogenesis in *zbtb16a/b* double mutants, it is possible that another mechanism contributes to the phenotype. Such a mechanism would be predicted to act before 24 hpf, when high levels of *zbtb16b* expression are present in the hindbrain. The possibility that Zbtb16 acts directly on proneural gene expression may be addressed in the future by the analysis of transcriptional targets of Zbtb16 in the hindbrain.

## MATERIALS AND METHODS

### Zebrafish lines

Wild-type *Danio rerio*, Tg[*egr2b:H2B-Citrine*] (Addison et al., 2018), *zbtb16a* single mutant and *zbtb16a*/*zbtb16b* double mutant (see below) embryos were produced by natural spawning and staged according to hpf and morphological criteria (Kimmel et al. 1995). *zbtb16a^−/−^*;*zbtb16b^−/−^* (*zbtb16a^−/−^*) embryos were produced by incrossing *zbtb16a^+/−^*;*zbtb16b^−/−^* (*zbtb16a^+/−^*) adults and *zbtb16a^+/−^*;*zbtb16b^−/−^* or *zbtb16a^+/+^*;*zbtb16b^−/−^* siblings were used as controls. The *zbtb16a* genotype of these embryos was confirmed post-staining by restriction fragment length polymorphism (RFLP) analysis of DNA isolated from individual embryos.

### Generation of the *zbtb16a/zbtb16b* mutant lines

TALEN constructs were designed to specifically target the *zbtb16a* and *zbtb16b* genes. Briefly, each TALEN array was designed according to the following criteria: (1) 16-20 base pair (bp) target sequence length; (2) a 14-17 bp spacer between the two arrays of the targeting pair; (3) presence of a thymine base immediately upstream of the target sequence; (4) absence of target sequence homology with other genes. A list of potential TALENs was generated using online software (https://boglab.plp.iastate.edu/; Cermak et al., 2011) and subsequent selection was performed manually.

TALEN construction was performed using the Golden Gate cloning technique designed for rapid generation of large constructs (Engler et al., 2009; Cermak et al., 2011). Plasmids were obtained from Addgene (Cat #1000000024) and TALENs were built using the 5-day protocol described by Cermak et al. (2011). Golden Gate-compatible destination vectors pCS2TAL3-DD and pCS2TAL3-RR (Dahlem et al., 2012) were obtained from Addgene (plasmids 37275 and 37276, respectively). Constructed plasmids were linearized with NotI restriction enzyme and capped mRNA was synthesized using the SP6 mMessage Machine Kit (Ambion). Equal amounts of left and right TALEN mRNA (100 pg each) were injected into one-cell stage wild-type zebrafish embryos.

The presence of TALEN-induced mutations was determined by High Resolution Melt (HRM) curve analysis of genomic DNA (gDNA) from 3 days postfertilization (dpf) embryos or fin clips from adult fish. Approximately 100 bp of gDNA was amplified around the target site. The primers used for amplification were 5′-GCCGTGTGGATTTCAGAGAC-3′ and 5′-GCGCATCTGATTAGCCTTGT-3′ for *zbtb16a*, and 5′-GTTCTGTGCGCATGAAACTC-3′ and 5′-CAGCCACCCTACAACTCTCC-3′ for *zbtb16b*. Triplicate 20 µl reactions containing 1 µl of gDNA solution were amplified and denatured in the presence of MeltDoctor HRM Dye (Applied Biosystems) using an Applied Biosystems 7900HT Fast Real-Time PCR System according to the manufacturer's instructions. HRM data were analysed using the Applied Biosystems HRM Software v2.0.

To sequence individual alleles, ∼500 bp of gDNA around the target site was amplified by PCR. The primers used for amplification were 5′-ACAAGAAAACGAACAACTGCAA-3′ and 5′-CTTGGAGCGTGGCAGTGTAG-3′ for *zbtb16a*, and 5′-CAGTTGCAGGAGCACTCAAG-3′ and 5′-AACCGCCATCTTGTATGGAA-3′ for *zbtb16b*. The PCR product was cloned into pGEM-T Easy (Promega), transformed into competent bacteria, and individual colonies were picked to carry out colony PCR using SP6 and T7 primers. The resulting PCR product was sequenced (GATC Biotech).

Embryos injected with TALENs targeting *zbtb16a* were grown to adulthood and outcrossed to wild-type fish in order to produce F1 embryos heterozygous for *zbtb16a* mutations. HRM analysis confirmed the presence of indel mutations in both F1 embryos and adults. Mutant alleles from F1 fish were sequenced and fish with an 8-bp deletion ∼50 bp downstream of the translation start site were used as founders for the *zbtb16a^+/−^* line, which was maintained in a heterozygous state as *zbtb16a^−/−^* fish do not reach adulthood.

Embryos injected with TALENs targeting *zbtb16b* were grown to adulthood and incrossed. HRM analysis confirmed the presence of indel mutations in F1 embryos. Heterozygous F1 adults were identified by RFLP analysis and mutant alleles were sequenced. Fish with identical 2-bp deletions at amino acid position 34 were incrossed to generate the *zbtb16b^−/−^* line, which was maintained in a homozygous state.

Double mutant fish were generated by crossing *zbtb16a^+/−^* fish to *zbtb16b^−/−^* fish. The double mutant line was maintained as *zbtb16a^+/−^*;*zbtb16b^−/−^*.

### Immunofluorescence analysis of zebrafish embryos

Embryos were grown to the desired stage and fixed in 4% paraformaldehyde (PFA) in phosphate-buffered saline (PBS) at room temperature for 2-3 h, dechorionated and washed several times in PBT (PBS + 0.1% Triton X-100) before blocking for 1-2 h at room temperature with 5% goat serum (GS) in PBT, 1% DMSO (10% GS for experiments described in Fig. 2). Primary antibodies were then added in 2% GS in PBT, 1% DMSO (5% GS for experiments described in Fig. 2) and embryos were incubated with gentle shaking overnight at 4° C. Embryos were washed extensively with PBT at room temperature and incubated overnight at 4°C with 2% GS in PBT, 1% DMSO (5% GS for experiments described in Fig. 2) containing the secondary antibodies. Embryos were counterstained with DAPI (1:1000) for 30 min at room temperature and washed extensively with PBT. For wholemount imaging, embryos were mounted on a glass slide in 70% glycerol and coverslipped. For transverse sections, embryos were embedded in 4% agarose and sections with a thickness of 80-120 μm were generated using a Leica VT1000 S Vibratome. Confocal *z*-stacks were acquired using a Leica SP5, Leica SP8 or Leica TCS SP2 confocal microscope.

A rabbit polyclonal antibody was raised against the purified BTB domain of the zebrafish Zbtb16a protein (Harlan Bioproducts). This antibody was subsequently found to recognize both Zbtb16a and Zbtb16b proteins (Fig. S1). The primary antibodies used for immunofluorescence in zebrafish embryos were: anti-Zbtb16 (rabbit IgG polyclonal; custom; Fig. S1, Fig. S2) at 1:500 or 1:2000 (Fig. 2), anti-HuC/D (mouse IgG_2b_; Molecular Probes, A-21272) at 1:200, anti-Myc (mouse IgG_1_; Santa Cruz Biotechnology, sc-40) at 1:500 and anti-GFAP (mouse IgG1; Dako, M076101-2) at 1:200. Secondary goat antibodies were Alexa Fluor conjugates (Thermo Fisher Scientific, A-11029, A-11037) used at 1:400 dilution.

### *In situ* hybridization

Embryos were fixed in 4% PFA for 4 h at room temperature, dehydrated in 100% methanol and stored at −20°C. Embryos were rehydrated through a series of 75%/50%/25% methanol in PBS and ISH was carried out as described previously (Xu et al., 1994). Colour development was carried out using 5-bromo-4-chloro-3-indolyl-phosphate (BCIP) and 4-nitro blue tetrazolium chloride (NBT). Double mutant and sibling control embryos from incrosses of *zbtb16a^+/−^* or *zbtb16a^+/−^*;*zbtb16b^−/−^* fish were always processed together and the genotype of individual embryos was confirmed after staining. For wholemount imaging, embryos were mounted on a glass slide in 70% glycerol and coverslipped. Images were acquired using a Zeiss Axioplan2 with an Axiocam HRc camera.

Two-colour fluorescent ISH was carried out using the Fast Blue/Fast Red detection system as previously described (Lauter et al., 2011). Detection of the fluorescent Fast Blue signal was carried by excitation with a 633 nm laser and detecting wavelengths greater than 650 nm. The Fast Red fluorescent signal was detected by excitation with a 561 nm laser and detecting wavelengths greater than 570 nm. Care was taken not to develop the Fast Blue signal for too long as the production of precipitate can obscure the subsequent development of the Fast Red signal.

For immunofluorescent staining following ISH, embryos were post-fixed in 4% PFA for 20 min at room temperature, washed several times in PBT, blocked for 2-3 h at room temperature with 5% GS in PBT, 1% DMSO, and incubated with primary and secondary antibodies as described above.

The following probes were generated from cDNA clones by *in vitro* transcription: *neurog1*, *neurod4*, *etv5b* (Gonzalez-Quevedo et al., 2010); *fgf3* (Breau et al., 2012); *fgf20b* (gift from A. Nechiporuk, Oregon Health and Science University, OR, USA); *hmx2* (Feng and Xu, 2010); *hoxb1a* and *hoxb2* (Prince et al., 1998). The template for the *zbtb16a* probe was generated by PCR using primers AGAAGATGACGAGGAGCGG and TTGCCACATAGCTCGCATC against the full-length cDNA clone (Sobieszczuk et al., 2010). The *fgf8* and *pax5* probe templates (based on Ma and Zhang, 2015) were generated from 28 hpf zebrafish cDNA by PCR. The reverse primers included a T7 promoter for direct *in vitro* transcription from the PCR product. Primers were: *fgf8* forward AATCCGGACCTACCAGCTTT; *fgf8* reverse GAAATTAATACGACTCACTATAGGCACATCCTGTGCTTCGCTTA; *pax5* forward TTGCGTCAGCAAGATACTGG; *pax5* reverse GAAATTAATACGACTCACTATAGGCGTGTGGCTGCGCTATAGTA.

### Hybridization chain reaction (HCR)

Embryos were raised to the desired stage, fixed in 4% PFA for 4 h at room temperature, dehydrated in 100% methanol and stored at −20°C. Embryos were rehydrated through a series of 75%/50%/25% methanol in PBS and HCR v3.0 was carried out following the standard protocol for zebrafish embryos (Choi et al., 2018). DAPI was added as a counterstain (1:1000 in 5×SSCT, 45 min at room temperature) and embryos were mounted in 70% glycerol for imaging. Confocal *z*-stacks were acquired using a Leica SP5 or Leica SP8 Falcon confocal microscope. All probes, amplifiers and buffers were purchased from Molecular Instruments. Profile plots were generated in ImageJ from sum projections of individual *z*-stacks. For 44 hpf, the ventral otic vesicle was excluded from the *z*-stack to avoid confounding effects on *etv5b* expression profiles. The area of measurement was specified by drawing a line parallel to the midline from the centre of r2 (identified based on morphology and gene expression) to r7 (Fig. S2A,B) and the width was adjusted to the width of the hindbrain. Grey values were extracted using the Plot Profile tool. Plotting was performed in Prism 9 and 3D reconstructions of *z*-stacks were generated in Imaris 9.3.1.

### RNAscope

RNAscope ISH (Wang et al., 2012a) was carried out using the RNAscope Multiplex Fluorescent v2 system as described previously (Wilcockson et al., 2022 preprint). The *fgf3* probes (850161-C4, ACDBio; gift from C. Hill, The Francis Crick Institute, UK) were detected by incubating the embryos with Multiplex FL V2 HRP-C4 for 15 min at 40°C, followed by incubation with tyramide (Sigma-Aldrich, T2879) coupled to fluorescein-NHS ester (Thermo Fisher Scientific, 46410) for 25 min in the dark at room temperature. Embryos were counterstained with DAPI (1:1000 in PBS+0.1% Tween) for 30 min at room temperature. Confocal *z*-stacks were acquired using a Leica SP8 Falcon confocal microscope.

### Inhibitor treatments

Embryos from *zbtb16a^+/−^*;*zbtb16b^−/−^* incrosses were grown to the 22 hpf stage, dechorionated manually and transferred to a six-well plate (∼30 embryos per well), then 3 ml of embryo medium containing either 2.5 μM SU5402 (Sigma-Aldrich, SML0443) or the equivalent volume of DMSO was added to each well. The embryos were incubated for 2 h at 28.5°C and fixed immediately after incubation for analysis by ISH. Sibling controls and double homozygous embryos were processed together and genotyped individually post-staining.

### Morpholino injections

MO were purchased from Gene Tools. Lyophilized stocks were dissolved in sterile water and stored at room temperature at a 1 mM concentration. For injections, stock solutions were diluted in 0.2 M KCl. We injected 1.8 nl of injection mixture containing the desired amount of each MO into the yolk of one- to four-cell-stage zebrafish embryos. *fgf3* MO was titrated to an amount that recapitulates the reduction of *pax5* staining in the otic vesicle of 24 hpf embryos described in *fgf3* morphants and genetic mutants (Leger and Brand, 2002; Hammond and Whitfield, 2011), while yielding a low number of malformed embryos. To minimize nonspecific effects, all MOs were co-injected with *p53* MO, which has been observed to block the activation of apoptotic pathways and to decrease the occurrence of hindbrain depression phenotypes (Robu et al., 2007; Gerety and Wilkinson, 2011). Morphologically abnormal embryos were excluded from analysis.

The MOs used in this study were: *fgf3* translation-blocking MO (5′-CATTGTGGCATGGCGGGATGTCGGC-3′; Maves et al., 2002; Maroon et al., 2002); *zbtb16b* translation-blocking MO (5′-ACATCAAGATTTACCGAACCATCTC-3′); *p53* MO (5′-GCGCCATTGCTTTGCAAGAATTG-3′); standard control MO (5′-CCTCTTACCTCAGTTACAATTTATA-3′).

### mRNA injections

Capped RNA was *in vitro* transcribed from linearized DNA using the SP6 mMessage mMachine kit (Ambion), precipitated with LiCl and dissolved in sterile water. Then 0.5 nl of solution containing the desired amount of each mRNA was injected into one cell of two-cell-stage zebrafish embryos. The constructs used to synthesize RNA were: pCS2-H2B-Citrine (gift from S. Megason, Harvard Medical School, MA, USA) and pCS2-MT-Zbtb16a (Sobieszczuk et al., 2010).

### Cell culture, transfection and immunohistochemistry

HEK293 cells were cultured as previously described (Poliakov et al., 2008) and transfected with 1 µg of the appropriate plasmid using FuGENE HD transfection reagent (Promega) according to the manufacturer's instructions. For immunofluorescence staining, cells were fixed 48 h post-transfection in 4% PFA for 15 min at room temperature, washed with PBST, blocked for 1 h in 4% GS and 2% bovine serum albumin, and stained with primary and secondary antibodies diluted in blocking buffer (Odyssey).

## Supplementary Material

10.1242/develop.201319_sup1 Supplementary information
