# Supplementary material for: Zbtb16 mediates a switch between Fgf signalling regimes in the developing hindbrain
Source: Development. 2023 Sep 14;150(18):dev201319. doi: 10.1242/dev.201319 (PMC10508701; doi:10.1242/dev.201319)
Supplement: Supplementary information [file develop-150-201319-s1.pdf]

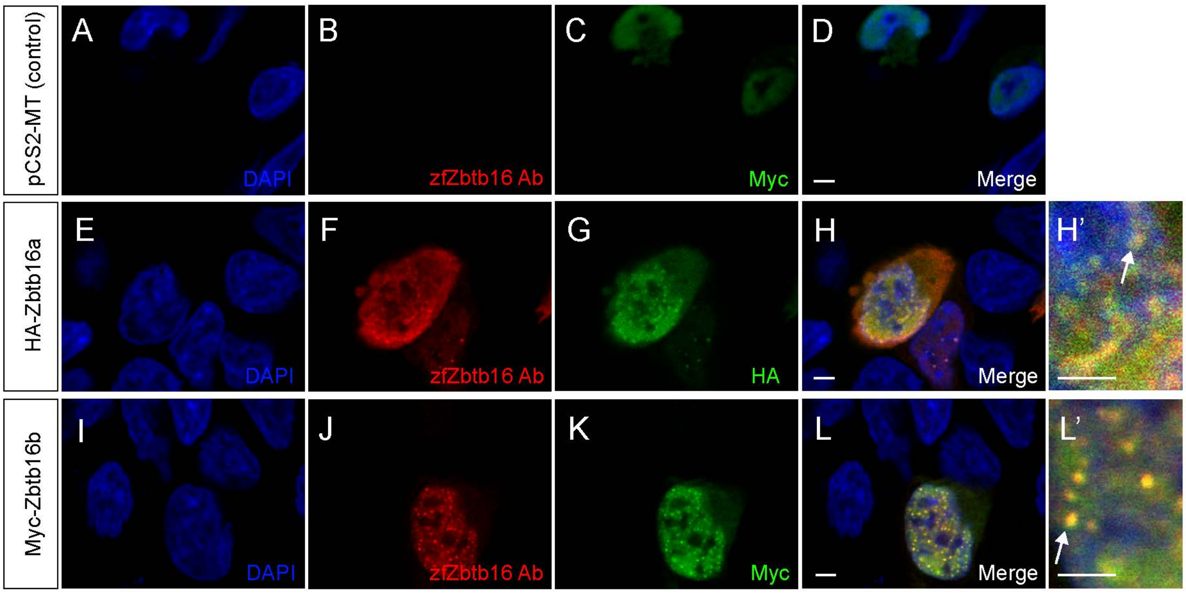

Fig. S1. An antibody raised against the Zbtb16a BTB domain recognises both Zbtb16 paralogues. Confocal images of HEK293 cells transfected with empty control vector (**A**-**D**), HA-tagged Zbtb16a (**E**-**H**) or Myc-tagged Zbtb16 (**I**-**L**) and stained with an antibody raised against the BTB domain of the zebrafish Zbtb16a protein (zfZbtb16) and antibodies against the HA/Myc tags. Arrows point to nuclear specks showing colocalization of the zfZbtb16 and anti-HA/anti-Myc signals. Scale bars: 5 μm.

Development • Supplementary information

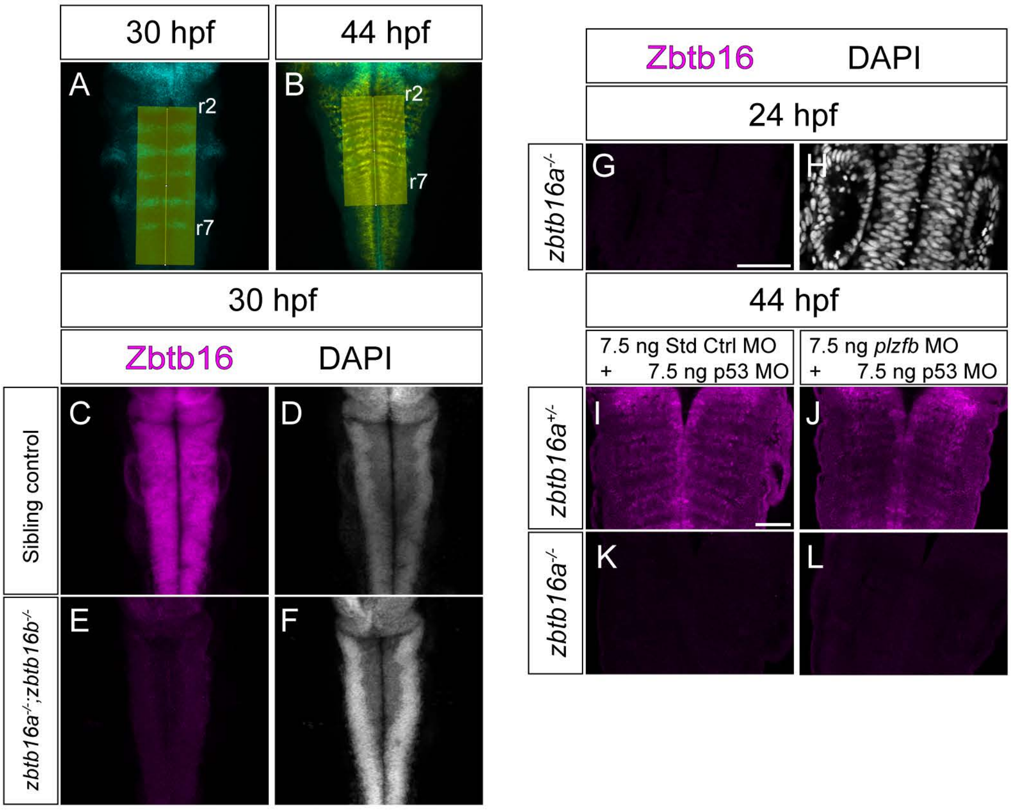

Fig. S2. Analysis of neurogenesis in zbtb16 mutants. (A,B) Demonstration of the procedure used for generating the profile plots shown in Fig. 3. Screenshots of area of measurement superimposed on sum projections through the Z-stack in ImageJ at 30 hpf (A) and 44 hpf (B). **(C-F)** anti-Zbtb16 staining in sibling control and *zbtb16a*$^{-/-}$;*zbtb16b*$^{-/-}$ embryos at 30 hpf. Sum projections through Z-stack. **(G,H)** anti-Zbtb16 immunofluorescent staining in *zbtb16a*$^{-/-}$ at 24 hpf. **(I-L)** anti-Zbtb16 immunofluorescent staining in 44 hpf *zbtb16a*$^{+/-}$ or *zbtb16a*$^{-/-}$ embryos injected with either control MO or plzfb MO. Slices from Z-stack. Scale bars: 50 μm.

Development • Supplementary information

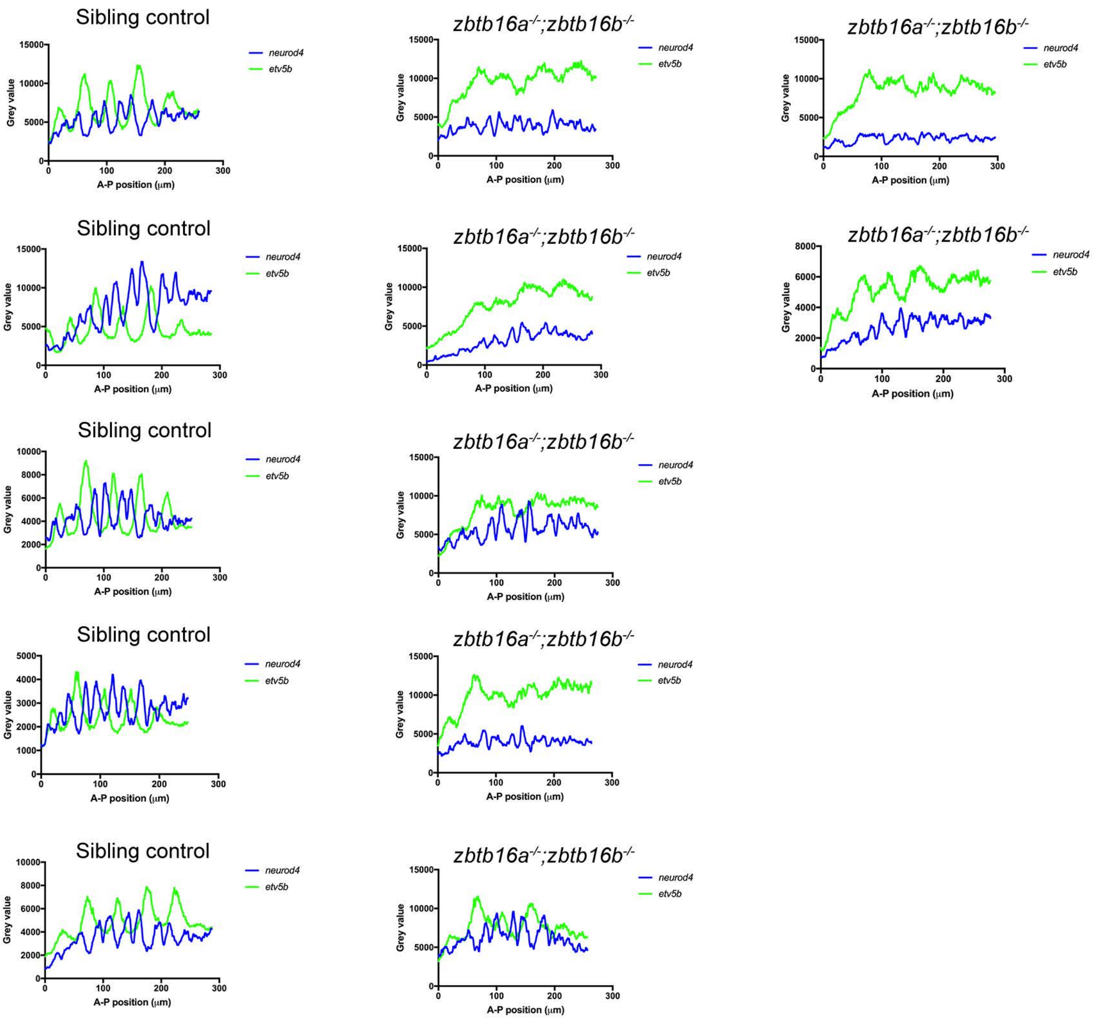

Fig. S3. Pattern of neurogenesis in *zbtb16* mutants. Profile plots of *etv5b* and *neurod4* detected by HCR in individual 30h *zbtb16a*$^{-/-}$;*zbtb16*$^{-/-}$ and sibling control *zbtb16a*$^{+/+}$;*zbtb16*$^{-/-}$ embryos, as shown in Fig. 3. Anterior to the left.

Development • Supplementary information

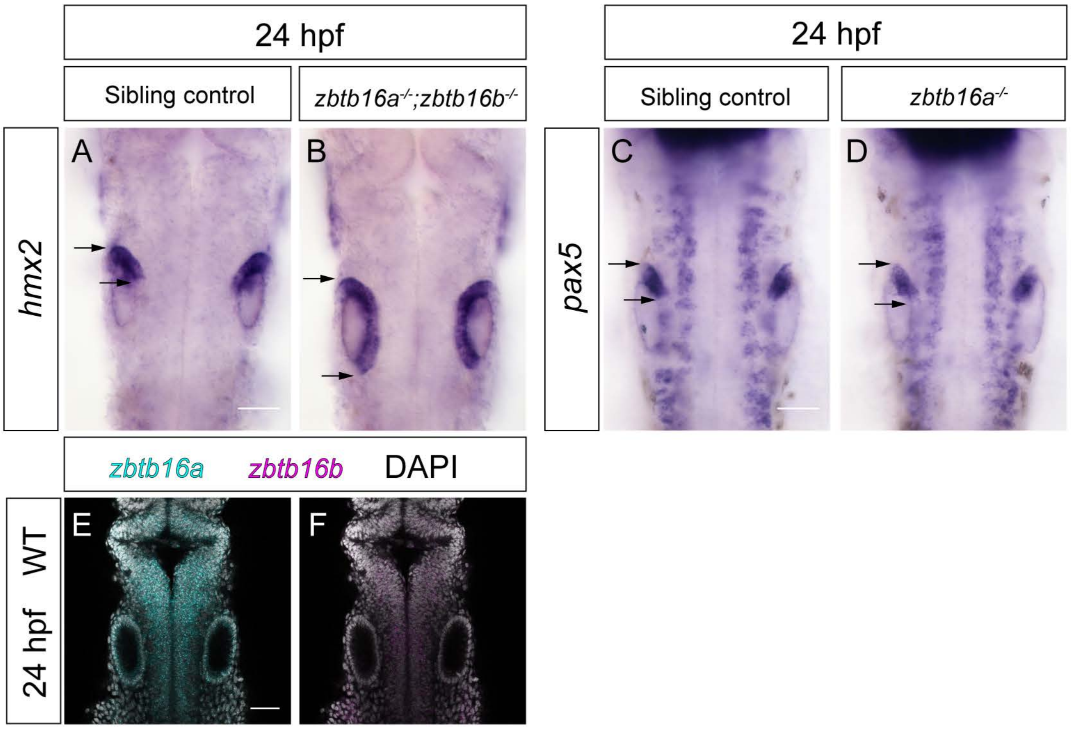

Fig. S4. Inner ear anteriorisation in *zbtb16a/b* double mutant but not in *zbtb16a* single mutant. (**A**,**B**) Colorimetric ISH for *hmx2* in 24 hpf sibling control (n=40) and *zbtb16a*$^{-/-}$;*zbtb16b*$^{-/-}$ (n=10) embryos. (**C**,**D**) ISH for *pax5* in 24 hpf sibling control (n=15) and *zbtb16a*$^{-/-}$ (n=9) embryos. Arrows in (A,B) and (E,F) show the antero-posterior extent of marker gene expression in the otic vesicle. (**E**,**F**) HCR for *zbtb16a* and *zbtb16b* in a wild type 24 hpf embryo; slices from confocal Z-stack. Scale bars: 50 μm

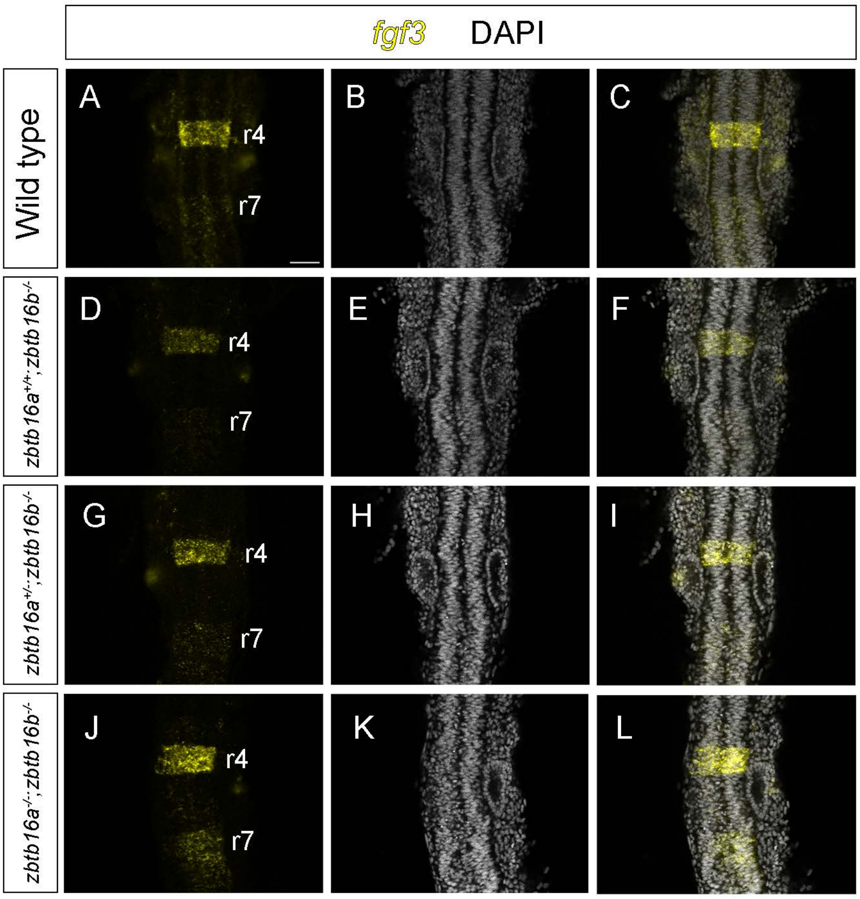

Fig. S5. Expression of *fgf3* at 16 hpf. RNAscope staining against *fgf3* in wild type (**A**-**C**), *zbtb16a*$^{+/+}$;*zbtb16*$^{-/-}$ (**D**-**F**), *zbtb16a*$^{+/-}$;*zbtb16*$^{-/-}$ (**G**-**I**) and *zbtb16a*$^{-/-}$; *zbtb16*$^{-/-}$ **(J-L)** embryos. Slices from confocal Z-stacks shown, anterior to the top. Abbreviations: r, rhombomere. Scale bar: 50 μm.

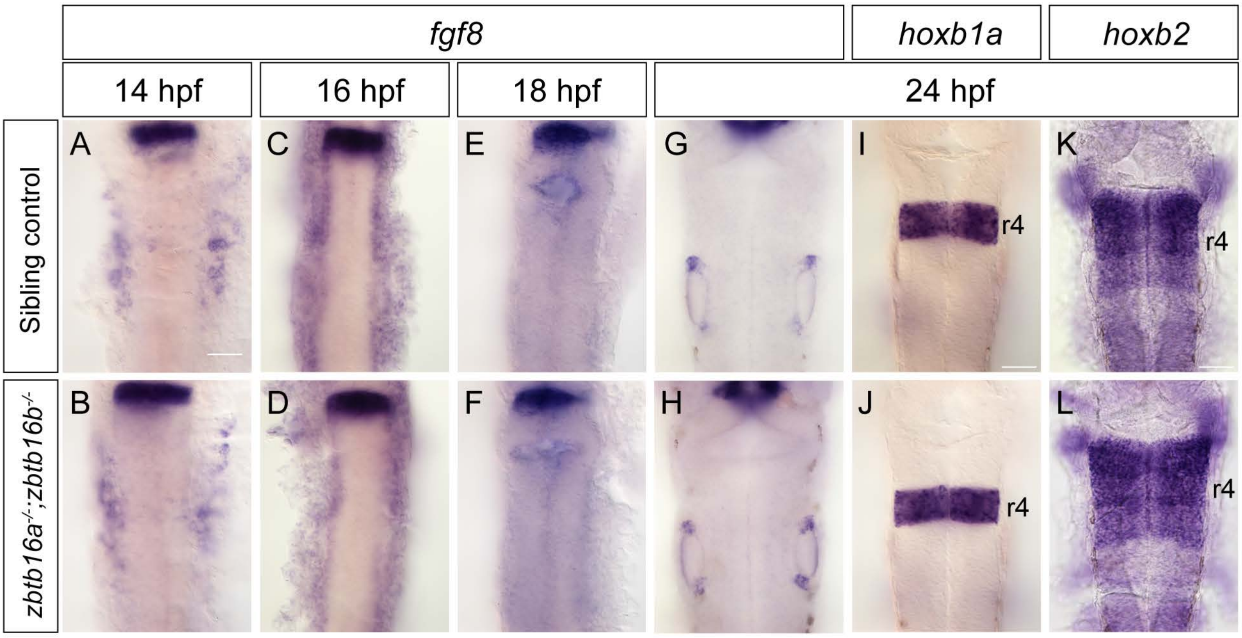

Fig. S6. Rhombomere identity is not compromised in *zbtb16* mutants. (**A-H**) ISH time-course of *fgf8* expression in the sibling control (14 hpf: n=39; 16 hpf; n=13; 18 hpf: n=16; 24hpf; n=35) and *zbtb16a*$^{-/-}$;*zbtb16b*$^{-/-}$ (14 hpf: n=8; 16 hpf: n=8; 18 hpf: n=5; 24 hpf: n=10) hindbrain. (**I, J**) Colorimetric ISH for *hoxb1a* in 24 hpf sibling control (n=33) and *zbtb16a*$^{-/-}$;*zbtb16b*$^{-/-}$ (n=6) embryos. (**K**, **L**) ISH for *hoxb2* in 24 hpf sibling control (n=22) and *zbtb16a*$^{-/-}$;*zbtb16b*$^{-/-}$ (n=7) embryos. Abbreviations: r, rhombomere. Scale bars: 50 µm.
