# Supplementary material for: Zbtb16 mediates a switch between Fgf signalling regimes in the developing hindbrain
Source: Development. 2023 Sep 14;150(18):dev201319. doi: 10.1242/dev.201319 (PMC10508701; doi:10.1242/dev.201319)
Supplement: Supplementary file 1 [file develop-150-201319_review_history.pdf]

Zbtb16 mediates a switch between Fgf signalling regimes in the developing hindbrain

Sami A. Leino, Sean C. J. Constable, Andrea Streit and David G. Wilkinson
DOI: 10.1242/dev.201319

Editor: Steve Wilson

Review timeline

Original submission:	24 September 2022
Editorial decision:	14 November 2022
First revision received:	1 June 2023
Editorial decision:	17 July 2023
Second revision received:	21 August 2023
Accepted:	22 August 2023

Original submission

First decision letter

MS ID#: DEVELOP/2022/201319

MS TITLE: Plzf mediates a switch between Fgf signalling regimes in the developing hindbrain

AUTHORS: Sami Leino, Sean C.J. Constable, Andrea Streit, and David G. Wilkinson

Apologies for the delay in obtaining reviews on your manuscript. I have now received all the referees' reports on the above manuscript, and have reached a decision. The referees' comments are appended below, or you can access them online: please go to BenchPress and click on the 'Manuscripts with Decisions' queue in the Author Area.

As you will see, the referees express interest in your work, but have some significant criticisms and many suggestions to improve your manuscript and strengthen the conclusions and insights you make. If you are able to revise the manuscript along the lines suggested, I will be happy receive a revised version of the manuscript. Please also note that Development will normally permit only one round of major revision. If it would be helpful, you are welcome to contact us to discuss your revision in greater detail. Please send us a point-by-point response indicating your plans for addressing the referees' comments, and we will look over this and provide further guidance.

Please attend to all of the reviewers' comments and ensure that you clearly highlight all changes made in the revised manuscript. Please avoid using 'Tracked changes' in Word files as these are lost in PDF conversion. I should be grateful if you would also provide a point-by-point response detailing how you have dealt with the points raised by the reviewers in the 'Response to Reviewers' box. If you do not agree with any of their criticisms or suggestions please explain clearly why this is so.

Reviewer 1

Advance summary and potential significance to field

Appears in the uploaded PDF

© 2023. Published by The Company of Biologists under the terms of the Creative Commons Attribution License (https://creativecommons.org/licenses/by/4.0/).

Comments for the author

This manuscript is attempting to describe a new role for Plzf in regulating the expression of different FGF ligands during hindbrain development, as it acts to coordinate a switch between early and late patterns and functions of FGFs. This switch appears to occur during hindbrain development in both fish and amniotes, granting it a particular significance, especially since so far it has not been fully resolved. As much as the subject of the manuscript is interesting and intriguing, there are certain elements and complementary data that are missing and should be fully addressed before granting publication.

In general- the manuscript's main conclusion is based on two main findings: (a) in the absence of Plzf at ~30 hpf, ETV5b expression spreads beyond rhombomere centers, and this phenomenon is coupled with the loss of striped-neuronal patterns (Fig.3), (b) the absence of Plzf at ~20hpf, FGf3 remains expressed at rh 4, as opposed to its expected downregulation at this stage (Fig.5). These findings led the authors to conclude that Plzf is involved in regulating the patterned neurogenic zones at ~ 40 hpf (Fig.2) by downregulating FGF3 expression a day earlier. Hence, in Plfz –nulls FGF3 and FGF20 are present during neural differentiation at the rhombomeres. Yet, the functional link that bonds the persistent expression of FGF3 at the earlier stages with the loss of organized neurogenesis later, is missing. This should be directly addressed as currently one cannot dissociate between earlier and later outcomes of the Plfz-KO. Some options are to over-express FGF3, expecting to show the same phenotypes as the Plfz-nulls, or alternatively to determine the effects of Plfz-KO in FGF3 mutants.

Several others issues that have to be addressed are indicated bellow:

1. Figure 1 presents the temporal and spatial expression pattern of Plzf paralogs at various stages, as the authors state that it has not been previously assessed. The figure relates to stages 14,16, and 24 hpf, however, as later stages are mostly discussed in this manuscript in regard to neural differentiation, this analysis should include those stages as well, all aligned together and not scattered in different figures as currently done.

2. In figure 2, it is unclear if Plzf is expressed at the neurogenic zones at stage 44 hpf, or at rh centers (where FGF20 is expressed). It appears from the GFAP-Plzf co-staining that it does complement with the neurogenic zones (2M-P) and it is stated in the text that cells in the HuC/D gaps are Plzf positive. However, transverse sections from the same stage shows HuC/D and Plzf expression at the same section in the mantle or ventricular zone, respectively. (2J-L). Whole mount image of Plzf expression at 44 hpf too small to conclude (2G).

3. In figure 3, Neurod4 expression appeared to be disorganized in the double mutant. However, based on the staining images and the HCR analysis, it seems like it somewhat decreased as a result of loss of Plzf, which might suggest reduced neurogenesis at this stage. This matter is neither addressed nor dismissed. This is an important point as the authors claim at their previous papers (i.e., Gonzalez-Quevedo et al., 2010) that FGF20 at rh centers regulates these patterns which are reduced in FGF20-nulls. This issue is confusing as the authors claim that Plfz KO leads to the same results as shown by FGF20 KO, yet FGF20 is unaffected in the Plfz null. Moreover, past studies by the Mason and Kimmel groups have shown that actually lack of both FGf3 and 8 at the hindbrain also leads to aberrant neurogenesis (but no effects are found by knocking down FGF3 only). This has to be discussed in the article. Notably, at their papers, a more thorough neurogenesis and axonogenesis analyses were provided compared to the current manuscript which only used NeuroD as a marker.

4. In figure 4, the authors show a broaden expression of ETV5b at 24 hpf of Plzf-nulls. Yet, the study by Maves et al (2202) has shown quite the opposite- erm expression is upregulated in the FGF3-morphants. This discrepancy must be addressed. Furthermore, the authors claims that a possible explanation for the ectopic ETV5b expression in the hindbrain could be an ectopic expression of several FGF ligands at this stage, resulting from loss of Plzf. They suggest that persistent expression of FGF3 at stages where it should have been downregulated, along with regular FGF20 expression (which is not affected by loss of Plzf) are possibly accounted for the ectopic ETV5b expression. ETV5b was reported in this manuscript (lines 244-247) as well as in previous work published from this lab (Gonzalez-Quevedo et al., 2010) as a target gene of FGF20. Is

© 2023. Published by The Company of Biologists under the terms of the Creative Commons Attribution License (https://creativecommons.org/licenses/by/4.0/).

it also a direct target gene of FGF3? If so, then the authors claim would make sense. Adding a spatial and temporal analysis of WT and mutant ETV5b expression in all the different stages, when FGF3 and FGF20 are in turn governing FGF functions, would help to understand this matter.

5. In the discussion the authors state that “Precise patterning of neurogenic zones by Fgf20 requires downregulation of FGF3 in the entire zebrafish hindbrain; upon loss of Plzf, *fgf3* persists and the ligand likely diffuses to the adjacent rhombomeres, as evidenced by ectopic *etv5b* in r3 of *plzfa-/- ;plzfb-/-* embryos”. Was patterning of neurogenic zones disrupted by the prolonged expression of FGF3? Authors reported an ectopic expression of neuroD4, although images are not very clear or convincing (Fig.3). This claim would benefit from analysis of expression of other markers of neurogenic zones and of axonal markers in the mutants (as done in previous FGF3/8 – knockdown studies in the zebrafish). This claim also needs to be demonstrated by unraveling further hindbrain markers in-between the earlier and later stages (as currently it is a too-big jump to conclude that due to the persistent expression of FGF3 at ~20 hpf, neurogenesis patterned are disturbed a day later).

6. Actually, in the inner ear data, the implication of persistent FGF3 on patterning has been elegantly done. Similar kind of analysis in continuation to Fig. 5 would greatly help to support the conclusions.

7. If indeed the persistent expression of FGF3 is the cause of the disrupted neuronal phenotype, the authors should consider replacing the order of the figures, by beginning with this finding.

Reviewer 2

Advance summary and potential significance to field

This paper describes analysis in zebrafish of expression and function of transcription factor genes plzfa and plzfb. Both genes show broad expression in the hindbrain during early segmentation stages, but showing zones of reduced expression in regions with elevated fgf3 expression. At later stages plzf genes become restricted to zones of neurogenesis flanking rhombomere boundaries, with prominent expression overlapping with neurod4. Embryos homozygous for knockout mutations in both plzf genes cause upregulation and prolongation of the early phase of expression of fgf3 in the hindbrain. Although double mutants show normal onset of expression of fgf20a and fgf20b in r-centers, the pattern of neurogenesis is grossly disturbed. Expression of neurod4 is lower than normal but is no longer spatially restricted. The altered pattern of neurogenesis is attributed to upregulation and prolongation of fgf3 expression in the hindbrain. Altered expression of fgf3 also correlates with transient anteriorization of the otic vesicle, as revealed by expanded expression of anterior otic markers pax5 and otx1. A major conclusion is that plzf genes are required for the transition from the early role of Fgf signaling in hindbrain patterning to the later role in establishing repeated patterns of neurogenesis around segment boundaries. The paper is well written, and figures are beautifully arranged and clear. Most of the authorsÂ’ conclusions are well supported by the data. The findings potentially provide important insight into the transition from early hindbrain pattern formation to spatially restricted neurogenesis. However, the analysis is somewhat incomplete and does not clearly delineate the regulatory relationships between plzf gene and fgf3 (or Fgf signaling in general). The full impact of the mutations on neurogenesis is also not fully addressed.
Additional experiments are needed to establish a fuller mechanistic understanding of plzf function.

Comments for the author

Major points:
1) The altered pattern of neurod4 in double mutants is striking, but it is not clear how progression of neurogenesis is altered. Is neurog1 also weak and unrestricted? Do mature neurons form at 30 hpf, or at later stages?
2) Does the early pattern of fgf3 eventually subside? The eventual restoration of proper expression of anterior otic genes suggests that it probably does subside soon after 24 hpf.
3) The authors argue that plzf genes repress fgf3 expression. However, the complementary patterns of fgf3 and plzf genes could reflect feedback as well. Does elevated Fgf signaling repress

© 2023. Published by The Company of Biologists under the terms of the Creative Commons Attribution License (https://creativecommons.org/licenses/by/4.0/).

plzf gene expression, and does SU5402 treatment cause upregulation of plzf expression? Along the same lines does the pattern of plzf transcription change in plzf double mutants?
4) A major conclusion of this paper is that defective neurogenesis seen in double mutants is caused by increased/expanded expression of fgf3. This should be tested in fgf3-/- mutants or fgf3 morphants.
Specifically, does disruption of fgf3 rescue the pattern of neurogenesis in double mutants?

Minor points:
According to ZFIN, the names of plzfa and plzfb have been changed to zbtb16a and zbtb16b. I appreciate that ongoing efforts to update nomenclature are potentially confusing, but the manuscript should adopt official nomenclature for the majority of the text, with brief reference to the older nomenclature in the Abstract and/or Introduction.

Reviewer 3

Advance summary and potential significance to field

The zinc finger Plzf proteins mediate a switch in Fgf signaling regimes in the zebrafish hindbrain. Its significance is not clear.

Comments for the author

In this report, the authors examine the role of a zinc finger transcription factors Plzfa and Plzfb in signaling events regulating neurogenesis in the zebrafish hindbrain. They describe Fgf signaling defects in plzfa; plzfb double mutants lacking Plzf function to conclude that Plzf mediates a switch in Fgf signaling regimes during hindbrain development. The data are of high quality and the conclusions are generally supported. However, additional experiments or data analysis are needed to strengthen the interpretations and conclusions. Some insight into how Plzfs could be mediating the switch in signaling regimes is needed to make a strong case for consideration in Development. The broader significance of the findings are unclear, weakening the report's impact.

Major Points
Figures 1 and 2:
In Fig. 1, while plzfb is downregulated in the anterior hindbrain between 14-24 hpf, plzfa expression and Plzf protein level in the hindbrain are relatively stable and ubiquitous. The authors do not propose a hypothesis for Plzf function; therefore, it is not clear what phenotype is expected in a Plzf-deficient condition (the double mutant). Similarly, in Fig. 2, while the complementary expression patterns of Plzf and HuC/D at 44 hpf are informative, they are not used to propose a hypothesis of Plzf function at that stage.

Double mutant analysis:
The end point for examining neurogenesis in plzf double mutant analysis is 30 hpf. Yet HuC and Plzf expression patterns in 30 hpf wild-type embryos were not described, making it difficult to interpret the double mutant phenotype. Conversely, the neurogenic pattern at 44 hpf is not described in the double mutant. While it is appreciated that the mutant phenotype at a specific age depends on gene expression (and function) at earlier stages, the absence of age-matched data on Plzf expression and neurogenesis patterns makes it difficult to interpret the mutant phenotype.

Line 194-196:
The loss of Plzf staining in double mutants must be shown.

Lines 268-275:
Fgf3 remains elevated in r4 in double mutants (negating the postulated Fgf switch), suggesting that Plzf normally suppresses fgf3. But fgf3 is highly expressed in r4 in 14 and 16 hpf inspite of strong plzfa (and Plzf) expression at these time points. Some insight into how Plzf affects fgf3 expression should be provided. Does Plzf directly regulate fgf3? Is the effect cell-autonomous? Does overexpression of plzfa suppress fgf3?
Figures 4 and 5:

© 2023. Published by The Company of Biologists under the terms of the Creative Commons Attribution License (https://creativecommons.org/licenses/by/4.0/).

Related to the issue of how Plzf regulates fgf3 (and etv5b) expression, why isn't the effect on etv5b expression mirroring fgf3 expression in double mutants? In 24 hpf double mutants, etv5b expression is only slightly elevated in the r4 region and not as strongly upregulated as would be expected from fgf3 expression. For example, etv5b and fgf3 expression at the mid-hindbrain boundary (MHB) seem superimposable in mutants but not in r4. Is it because fgf8 is also expressed at MHB? Cell autonomy or GOF experiments may provide insight into this issue as well.

Lines 286-287:
Since fgf3 is greatly upregulated in r4 in double mutants, there should be reduced neurogenesis in this region by 44 hpf. Is this the case?

Lines 295-334:
While this section presents nice data on inner ear patterning defects in double mutants, it is not relevant to the main findings of the report or its significance. This section should be shortened or removed.

Lines 336-485:
The discussion section is too long for the amount of functional data presented and the significance of the findings. Sections on comparative function of Plzf in amniotes and fish, and on Plzf in neural progenitors should be greatly truncated.

Lines 425-428:
This statement suggests that the effect on neurogenesis in double mutants could be non-autonomous. Such an experiment would be worthwhile and insightful and greatly strengthen this report.

Minor Points
Lines 200-202, 206-208:
The phenotype at 30 hpf could result from an earlier patterning defect. Although Fig. 5 shows fgf3 expression in r4 in mutants, it is important to better document normal hindbrain patterning at 14-18 hpf in double mutants using rhombomere markers like hoxa2, egr2 and mafB. Related to this, based on extensive Plzf expression at 14-16 hpf (and perhaps at earlier stages) shown in Fig. 1, can you state definitively that there is no early role for plzf genes in hindbrain development?

Line 208:
While the procedure for generating the plots in Figs. 3I and S2 is described in the Methods section, it is unclear. The "center of rhombomere 2" is defined as AP position = 0. What is the "center of r2"? The values seem to be measures of the total signal in a rectangular area and not a line scan. It would be very helpful to place a schematic panel describing the measurement in Fig. S2 (and cite in Methods).

Lines 211-212:
Is the spatial pattern of neurogenesis disrupted in mutants due to an increase in neuron number or due to more scattering of neurons into rhombomere centers? Counts of neurons in the two conditions would help address this.

Lines 227-229, 290-291:
While the texts suggest that the anterior hindbrain is relatively unaffected in mutants, Figs. 3E and 4J indicate otherwise. In situs with rhombomere markers at 14-18 hpf (see earlier point) can address this issue definitively.

Lines 399-402:
Plzf expression is not rhombomere-specific in zebrafish between 14-24 hpf (Figs. 1 and 2), maybe only at 44 hpf (Fig. 2). Similarly, fgf3 expression is not anticorrelated with Plzf expression at 14-18 hpf, where high levels of fgf3 persist in the presence of high Plzf expression (Figs. 1 and 4). Please rewrite these sentences.

© 2023. Published by The Company of Biologists under the terms of the Creative Commons Attribution License (https://creativecommons.org/licenses/by/4.0/).

First revision

Author response to reviewers' comments

We thank the reviewers for their insightful and constructive comments on the manuscript. We have carried out further experiments and made extensive modifications to the text to address the points raised, as detailed below. These changes provide further support for the conclusions of the study and have significantly improved the manuscript.

As pointed out by a reviewer, Zbtb16 has become the official name in zebrafish, and we have altered the manuscript text accordingly, but for consistency within our response we continue to use Plzf below.

Reviewer 1

This manuscript is attempting to describe a new role for Plzf in regulating the expression of different FGF ligands during hindbrain development, as it acts to coordinate a switch between early and late patterns and functions of FGFs. This switch appears to occur during hindbrain development in both fish and amniotes, granting it a particular significance, especially since so far it has not been fully resolved. As much as the subject of the manuscript is interesting and intriguing, there are certain elements and complementary data that are missing and should be fully addressed before granting publication.

In general- the manuscript's main conclusion is based on two main findings: (a) in the absence of Plzf at ~30 hpf, ETV5b expression spreads beyond rhombomere centers, and this phenomenon is coupled with the loss of striped-neuronal patterns (Fig.3), (b) the absence of Plzf at ~20hpf, FGf3 remains expressed at rh 4, as opposed to its expected downregulation at this stage (Fig.5). These findings led the authors to conclude that Plzf is involved in regulating the patterned neurogenic zones at ~ 40 hpf (Fig.2) by downregulating FGF3 expression a day earlier. Hence, in Plfz –nulls FGF3 and FGF20 are present during neural differentiation at the rhombomeres. Yet, the functional link that bonds the persistent expression of FGF3 at the earlier stages with the loss of organized neurogenesis later, is missing. This should be directly addressed as currently one cannot dissociate between earlier and later outcomes of the Plfz-KO. Some options are to over-express FGF3, expecting to show the same phenotypes as the Plfz-nulls, or alternatively to determine the effects of Plfz-KO in FGF3 mutants.

Answer: This is a good point, and we agree that further work is needed to link the relationship between Plzf and Fgf3 to the phenotypes that we observe in the Plzf double mutant. As suggested by the reviewer, one approach is to analyse the effect of loss of Fgf3 function on the altered patterning of neurogenesis in Plzf double mutants. We have carried out Fgf3 knockdowns in Plzf double mutants and find that there is partial rescue of the altered pattern of etv5 expression (new Fig.6). This supports the main conclusion of the study: that Plzf is required to downregulate the early segmental expression of fgf3. However, we have not detected a consistent rescue of the organisation of neurogenesis after fgf3 knockdown in Plzf double mutants. A potential explanation is that the knockdown of fgf3 is incomplete and variable, and that residual fgf3 signalling is sufficient to affect the patterning of neurogenesis.

As another approach to dissect the contribution of early versus late Plzf function, we have obtained evidence for stage-specific expression of the plzfa and plzfb paralogues. Immunodetection reveals that Plzf protein is undetectable in the hindbrain from 24 hpf onwards in plzfa mutants, correlating with the observation that plzfb mRNA has been downregulated at these stages. Thus, in the hindbrain both plzfa and plzfb contribute to Plzf protein at early stages, whereas from 24 hpf onwards only plzfa contributes. Since loss of both paralogues is required for the downregulation of fgf3 expression and pattern of neurogenesis, this suggests that the effects on Fgf3 expression and neurogenic patterning are due to roles of plzf genes at early stages.

We have now analysed fgf3 expression in Plzf double mutant embryos at later stages and find that the segmental expression in r4, r6 and r7 persists until 36 hpf, but by 44 hpf is at very low levels (new Fig.5). The persistence of fgf3 expression in the double mutant thus overlaps in time with the altered patterning of neurogenesis at 30 hpf. We have now analysed neurogenic markers at 44 hpf

© 2023. Published by The Company of Biologists under the terms of the Creative Commons Attribution License (https://creativecommons.org/licenses/by/4.0/).

in the double mutant and find that by this stage the pattern of neurogenesis is no longer disrupted (new Fig.3).

Several others issues that have to be addressed are indicated below:
1. Figure 1 presents the temporal and spatial expression pattern of Plzf paralogs at various stages, as the authors state that it has not been previously assessed. The figure relates to stages 14,16, and 24 hpf, however, as later stages are mostly discussed in this manuscript in regard to neural differentiation, this analysis should include those stages as well, all aligned together and not scattered in different figures as currently done.

Answer: We have now added HCR expression data for the plzf paralogues at 30 and 44 hpf to this Figure.

2. In figure 2, it is unclear if Plzf is expressed at the neurogenic zones at stage 44 hpf, or at rh centers (where FGF20 is expressed). It appears from the GFAP-Plzf co-staining that it does complement with the neurogenic zones (2M-P) and it is stated in the text that cells in the HuC/D gaps are Plzf positive. However, transverse sections from the same stage shows HuC/D and Plzf expression at the same section in the mantle or ventricular zone, respectively. (2J-L). Whole mount image of Plzf expression at 44 hpf too small to conclude (2G).

Answer: We have modified the text to better explain the expression pattern of Plzf. As stated in the Results, Plzf is present throughout the ventricular zone, and thus is both in the neurogenic zones and at rhombomere centres. This is seen in the side views of Fig.2M,T. At 44 hpf, the neurogenic zones comprise radial glial cells with fibres extending into the mantle zone, and differentiating neurons migrate along these fibres. Plzf is being downregulated in the differentiating neurons migrating along the fibres. This is seen in the side views in Fig.2 M, T. The glial fibres physically exclude the neurons detected by HuC/D staining, thus creating the complementarity between Plzf and HuC/D seen in side views (Fig.2M-P). The lack of clarity may have arisen from the coronal confocal sections that show Plzf in stripes complementary to HuC/D (Fig.2G-I). We have now explained that the plane of section is below the level of the ventricular zone and thus is passing through the glial fibres within the mantle zone.

3. In figure 3, Neurod4 expression appeared to be disorganized in the double mutant. However, based on the staining images and the HCR analysis, it seems like it somewhat decreased as a result of loss of Plzf, which might suggest reduced neurogenesis at this stage. This matter is neither addressed nor dismissed. This is an important point as the authors claim at their previous papers (i.e., Gonzalez-Quevedo et al., 2010) that FGF20 at rh centers regulates these patterns which are reduced in FGF20-nulls. This issue is confusing as the authors claim that Plfz KO leads to the same results as shown by FGF20 KO, yet FGF20 is unaffected in the Plfz null. Moreover, past studies by the Mason and Kimmel groups have shown that actually lack of both FGf3 and 8 at the hindbrain also leads to aberrant neurogenesis (but no effects are found by knocking down FGF3 only). This has to be discussed in the article. Notably, at their papers, a more thorough neurogenesis and axonogenesis analyses were provided compared to the current manuscript which only used NeuroD as a marker.

Answer: We thank the reviewer for pointing out the decrease in neurod4 expression detected in the HCR stainings and quantifications. This is consistent with the inhibitory effect of Fgf signalling on neurogenesis, and we now discuss this and have carried out quantifications at other stages.

We did not intend to imply that Plzf mutants have the same results as Fgf20 mutants and have clarified this in the revised text. The similarity is that in both mutants the patterning of neurogenesis is less organised than in wild type embryos in which neurogenesis is confined to zones adjacent to hindbrain boundaries. In fgf20 mutants, there is an absence of Fgf signalling at late stages in the hindbrain and thus a lack of inhibition at rhombomere centres. In Plzf mutants, the persistent expression of Fgf3 leads to widespread Fgf signalling i.e. Fgf signalling is no longer confined to segment centres.

As mentioned in response to a later point, we have now analysed neurog1 expression as a further marker of neurogenesis.

© 2023. Published by The Company of Biologists under the terms of the Creative Commons Attribution License (https://creativecommons.org/licenses/by/4.0/).

Regarding the studies of the Mason and Kimmel group, these revealed the key roles of Fgf3 and Fgf8 in early segmental patterning of the hindbrain, in which their expression in r4 is required for segmentation of the adjacent regions. The changes in neurogenesis and axons were informative markers of the altered segmental identities after loss of Fgf3/Fgf8 signalling. However, this is a qualitatively different situation from the persistence of Fgf3 signalling found in the current study. We have not carried out an analysis of axonal patterns as this is a later aspect of neuronal development than the patterning of neurogenesis which is the focus of this study.

4. In figure 4, the authors show a broaden expression of ETV5b at 24 hpf of Plzf-nulls. Yet, the study by Maves et al (2202) has shown quite the opposite- erm expression is upregulated in the FGF3-morphants. This discrepancy must be addressed. Furthermore, the authors claims that a possible explanation for the ectopic ETV5b expression in the hindbrain could be an ectopic expression of several FGF ligands at this stage, resulting from loss of Plzf. They suggest that persistent expression of FGF3 at stages where it should have been downregulated, along with regular FGF20 expression (which is not affected by loss of Plzf) are possibly accounted for the ectopic ETV5b expression. ETV5b was reported in this manuscript (lines 244- 247) as well as in previous work published from this lab (Gonzalez-Quevedo et al., 2010) as a target gene of FGF20. Is it also a direct target gene of FGF3? If so, then the authors claim would make sense. Adding a spatial and temporal analysis of WT and mutant ETV5b expression in all the different stages, when FGF3 and FGF20 are in turn governing FGF functions, would help to understand this matter.

Answer: Previous studies have found that etv5/erm is a general target of Fgf signalling. Consistent with this, Maves et al find that erm expression in the early hindbrain is lost following double knockdown of fgf3 and fgf8, and Raible and Brand (Mech Dev 2001) report upregulation of erm following overexpression of fgf3. Investigation of why Maves et al found that erm is upregulated after fgf3 knockdown is beyond the scope of the current study, but their data are consistent with compensatory increased expression of fgf8. We have not included a further figure to document the expression of etv5 at different stages in the hindbrain as this has been well-established in previous studies: Fgf3/8 regulate the early widespread expression, and Fgf20 regulates the later expression localised to rhombomere centres.

5. In the discussion the authors state that “Precise patterning of neurogenic zones by Fgf20 requires downregulation of FGF3 in the entire zebrafish hindbrain; upon loss of Plzf, *fgf3* persists and the ligand likely diffuses to the adjacent rhombomeres, as evidenced by ectopic *etv5b* in r3 of *plzfa-/-;plzfb-/-* embryos”. Was patterning of neurogenic zones disrupted by the prolonged expression of FGF3? Authors reported an ectopic expression of neuroD4, although images are not very clear or convincing (Fig.3). This claim would benefit from analysis of expression of other markers of neurogenic zones and of axonal markers in the mutants (as done in previous FGF3/8 –knockdown studies in the zebrafish). This claim also needs to be demonstrated by unraveling further hindbrain markers in-between the earlier and later stages (as currently it is a too-big jump to conclude that due to the persistent expression of FGF3 at ~20 hpf, neurogenesis patterned are disturbed a day later).

Answer: We have carried out further analyses to show that the patterning of neurogenesis is disrupted in the plzf double mutants. We had chosen neurod4 as the most informative marker since it is upregulated early following the onset of the neurogenic cascade that underlies differentiation, so gives a clear picture of the organisation of neurogenesis. We now include data for neurog1 which is at the top of the neurogenic cascade, but also expressed in progenitors either at lower levels or oscillating. Axonal markers would not be informative for the organisation of neurogenesis; differentiating neurons migrate along the glial fibres into the mantle zone where they are repositioned. Our further analysis reveals that the persistent expression of fgf3 continues to 36 hpf, so there is no longer the difficulty that this appears to be well before the neurogenesis phenotype.

6. Actually, in the inner ear data, the implication of persistent FGF3 on patterning has been elegantly done. Similar kind of analysis in continuation to Fig. 5 would greatly help to support the conclusions.

© 2023. Published by The Company of Biologists under the terms of the Creative Commons Attribution License (https://creativecommons.org/licenses/by/4.0/).

Answer: We are unsure what is being asked for here, but our further analysis described above suggests that plzfa and plzfb are required at early and not late stages for downregulation of Fgf3 in the hindbrain.

7. If indeed the persistent expression of FGF3 is the cause of the disrupted neuronal phenotype, the authors should consider replacing the order of the figures, by beginning with this finding.

Answer: We prefer to keep the current order as it reflects the logical flow of the study: the generation of mutants to test the role of Plzf in the regulation of neurogenesis suggested by previous studies; finding of altered patterns of neurogenesis; finding of altered Fgf signalling that might explain this; demonstration that early Fgf3 expression persists in Plzf double mutants.

Reviewer 2 Advance Summary and Potential Significance to Field...

This paper describes analysis in zebrafish of expression and function of transcription factor genes plzfa and plzfb. Both genes show broad expression in the hindbrain during early segmentation stages, but showing zones of reduced expression in regions with elevated fgf3 expression. At later stages plzf genes become restricted to zones of neurogenesis flanking rhombomere boundaries, with prominent expression overlapping with neurod4. Embryos homozygous for knockout mutations in both plzf genes cause upregulation and prolongation of the early phase of expression of fgf3 in the hindbrain. Although double mutants show normal onset of expression of fgf20a and fgf20b in r-centers, the pattern of neurogenesis is grossly disturbed. Expression of neurod4 is lower than normal but is no longer spatially restricted. The altered pattern of neurogenesis is attributed to upregulation and prolongation of fgf3 expression in the hindbrain. Altered expression of fgf3 also correlates with transient anteriorization of the otic vesicle, as revealed by expanded expression of anterior otic markers pax5 and otx1. A major conclusion is that plzf genes are required for the transition from the early role of Fgf signaling in hindbrain patterning to the later role in establishing repeated patterns of neurogenesis around segment boundaries. The paper is well written, and figures are beautifully arranged and clear. Most of the authors' conclusions are well supported by the data. The findings potentially provide important insight into the transition from early hindbrain pattern formation to spatially restricted neurogenesis. However, the analysis is somewhat incomplete and does not clearly delineate the regulatory relationships between plzf gene and fgf3 (or Fgf signaling in general). The full impact of the mutations on neurogenesis is also not fully addressed. Additional experiments are needed to establish a fuller mechanistic understanding of plzf function.

Reviewer 2 Comments for the Author...

Major points:
1) The altered pattern of neurod4 in double mutants is striking, but it is not clear how progression of neurogenesis is altered. Is neurog1 also weak and unrestricted? Do mature neurons form at 30 hpf, or at later stages?

Answer: We now include data for neurog1. Neurog1 is expressed in all progenitors (where it has important functions) and studies by other labs suggest that it switches from low level or oscillating expression to sustained expression at the onset of neuronal differentiation. Consequently, unlike neurod4, the expression of neurog1 is not strongly patterned in the zebrafish hindbrain. We have not analysed later steps of neuronal differentiation.

2) Does the early pattern of fgf3 eventually subside? The eventual restoration of proper expression of anterior otic genes suggests that it probably does subside soon after 24 hpf.

Answer: This is an important point and we have now analysed later stages. We find that the expression of fgf3 persists in r4, r6 and r7 to 36 hpf, and thus overlaps in time with the disrupted organisation of neurogenesis. Only low levels of Fgf3 expression are found at 44 hpf, when the neurogenic pattern is restored to normal.

© 2023. Published by The Company of Biologists under the terms of the Creative Commons Attribution License (https://creativecommons.org/licenses/by/4.0/).

3) The authors argue that plzf genes repress fgf3 expression. However, the complementary patterns of fgf3 and plzf genes could reflect feedback as well. Does elevated Fgf signaling repress plzf gene
expression, and does SU5402 treatment cause upregulation of plzf expression? Along the same lines, does the pattern of plzf transcription change in plzf double mutants?

Answer: We thank the reviewer for these interesting questions on how Plzf itself is regulated. We have not had time to pursue these ideas as we have focussed on firming up the steps downstream of Plzf to the organisation of Fgf signalling and neurogenesis. We now mention these questions in the Discussion.

4) A major conclusion of this paper is that defective neurogenesis seen in double mutants is caused by increased/expanded expression of fgf3. This should be tested in fgf3-/- mutants or fgf3 morphants. Specifically, does disruption of fgf3 rescue the pattern of neurogenesis in double mutants?

Answer: We have carried out Fgf3 knockdowns in Plzf double mutants and find that there is partial rescue of the altered pattern of etv5 expression (new Fig.6). This supports the main conclusion of the study: that Plzf is required to downregulate the early segmental expression of fgf3. However, we have not detected a consistent rescue of the organisation of neurogenesis after fgf3 knockdown in Plzf double mutants. A potential explanation is that the knockdown of fgf3 is incomplete and variable, and that residual fgf3 signalling is sufficient to affect the patterning of neurogenesis. Previous work has shown that Fgf signalling inhibits neurogenesis at the relevant stages (Gonzalez-Quevedo et al, 2010) and this is consistent with correlative evidence in the current study: (i) the patterning and levels of proneural genes are closer to normal in the 44 hpf double mutant, which has lower levels of *fgf3* compared to previous stages, and (ii) at 30 hpf, double homozygous embryos with more normally patterned *etv5b* expression also show better patterning of *neurod4* (Fig. S2 with the profile plots).

Minor points:
According to ZFIN, the names of plzfa and plzfb have been changed to zbtb16a and zbtb16b. I appreciate that ongoing efforts to update nomenclature are potentially confusing, but the manuscript should adopt official nomenclature for the majority of the text, with brief reference to the older nomenclature in the Abstract and/or Introduction.

Answer: We have switched from Plzf to Zbtb16 throughout the manuscript and figures.

Reviewer 3 Comments for the Author...

In this report, the authors examine the role of a zinc finger transcription factors Plzfa and Plzfb in signaling events regulating neurogenesis in the zebrafish hindbrain. They describe Fgf signaling defects in plzfa; plzfb double mutants lacking Plzf function to conclude that Plzf mediates a switch in Fgf signaling regimes during hindbrain development. The data are of high quality and the conclusions are generally supported. However, additional experiments or data analysis are needed to strengthen the interpretations and conclusions. Some insight into how Plzfs could be mediating the switch in signaling regimes is needed to make a strong case for consideration in Development. The broader significance of the findings are unclear, weakening the report's impact.

Answer: The broader significance of the manuscript is that many signalling pathways have stage-specific expression and functions, and this requires that early patterns of expression are downregulated by the time that later expression is acting. The specific mechanism used to achieve this is likely different in different tissues. Here, we uncover such a mechanism for Fgf family members in the developing hindbrain. It may be significant that Plzf has also been linked to regulation of Fgf signalling in the chick and mouse spinal cord, but through a different mechanism-regulation of Fgf receptor levels - suggestive of diverse links between Plzf and Fgf signalling.

Major Points

© 2023. Published by The Company of Biologists under the terms of the Creative Commons Attribution License (https://creativecommons.org/licenses/by/4.0/).

Figures 1 and 2:
In Fig. 1, while plzfb is downregulated in the anterior hindbrain between 14-24 hpf, plzfa expression and Plzf protein level in the hindbrain are relatively stable and ubiquitous. The authors do not propose a hypothesis for Plzf function; therefore, it is not clear what phenotype is expected in a Plzf-deficient condition (the double mutant). Similarly, in Fig. 2, while the complementary expression patterns of Plzf and HuC/D at 44 hpf are informative, they are not used to propose a hypothesis of Plzf function at that stage.

Answer: We have modified the beginning of the Results section to clarify that the hypothesis is not derived from the expression pattern of Plzf or relationship between Plzf and HuC/D expression. The hypothesis comes from our previous work (Sobieszczuk et al 2010) suggesting that Plzf inhibits neurogenic gene expression. The prediction is that loss of Plzf function will lead to excessive and/or premature neurogenesis. To test this we generated null mutants. Unexpectedly, we instead found reduced and disorganised neurogenesis. The interpretation of the findings required a more detailed analysis of the expression pattern of plzf, so we present this first in the manuscript.

Double mutant analysis:
The end point for examining neurogenesis in plzf double mutant analysis is 30 hpf. Yet HuC and Plzf expression patterns in 30 hpf wild-type embryos were not described, making it difficult to interpret the double mutant phenotype. Conversely, the neurogenic pattern at 44 hpf is not described in the double mutant. While it is appreciated that the mutant phenotype at a specific age depends on gene expression (and function) at earlier stages, the absence of age-matched data on Plzf expression and neurogenesis patterns makes it difficult to interpret the mutant phenotype.

Answer: We now include data for plzf paralogues at 30 hpf and 44 hpf (new Fig.1). HuC expression at 30 hpf is not relevant to the interpretations - as at 44 hpf it fills the mantle zone except where excluded by glial fibres. At all stages, Plzf expression occurs throughout the ventricular zone and in differentiating neurons at early stages of their migration into the mantle zone. In revised Fig.3 we now show further data and analysis of the organisation of neurogenesis at 30 hpf, and include data for 44 hpf.

Line 194-196:
The loss of Plzf staining in double mutants must be shown.

Answer: We now show these data both for double plzfa/plzfb and single plzfa mutants in Fig. S3.

Lines 268-275:
Fgf3 remains elevated in r4 in double mutants (negating the postulated Fgf switch), suggesting that Plzf normally suppresses fgf3. But fgf3 is highly expressed in r4 in 14 and 16 hpf in spite of strong plzfa (and Plzf) expression at these time points. Some insight into how Plzf affects fgf3 expression should be provided. Does Plzf directly regulate fgf3? Is the effect cell-autonomous? Does overexpression of plzfa suppress fgf3?

Answer: We have added panels to Fig.1 to clarify that there is low level Plzf expression in the posterior hindbrain at 14 hpf, which has been upregulated from 16 hpf onwards. The increase in Plzf expression thus precedes the progressive downregulation of fgf3 expression from 16-24 hpf.

These are excellent suggestions for linking Plzf to fgf3 gene regulation. Due to time constraints we were not able to carry out experiments to test direct regulation or cell autonomy. We have now carried out experiments to test the effect of overexpressing Plzf by injection of mRNA into one half of the embryo (new Fig.7). We find that Plzf overexpression leads to a major decrease in Fgf3 expression compared with the uninjected half, supporting that Plzf acts upstream of Fgf3 to downregulate its expression.

Figures 4 and 5:
Related to the issue of how Plzf regulates fgf3 (and etv5b) expression, why isn't the effect on etv5b expression mirroring fgf3 expression in double mutants? In 24 hpf double mutants, etv5b expression is only slightly elevated in the r4 region and not as strongly upregulated as would be expected from fgf3 expression. For example, etv5b and fgf3 expression at the mid-hindbrain boundary (MHB) seem

© 2023. Published by The Company of Biologists under the terms of the Creative Commons Attribution License (https://creativecommons.org/licenses/by/4.0/).

superimposable in mutants but not in r4. Is it because fgf8 is also expressed at MHB? Cell autonomy or GOF experiments may provide insight into this issue as well.

Answer: The reviewer correctly observes that there is not a tight spatial correlation between fgf3 expression and etv5b. However, this is also the case in wild type embryos, where previous studies found that etv5b expression occurs in a broad domain in the hindbrain, without major differences in level in r4 and adjacent rhombomeres. This is consistent with the evidence that Fgf3 acts on adjacent rhombomeres during segmentation, and thus may be diffusing away from r4. A possible explanation for the broad expression of etv5b is that the level of Fgf activation in the region flanking r4 is enough to induce full expression. Another possible factor is that there is feedback inhibition of the Fgf pathway through Sprouty genes that may suppress initial high level activation.

Lines 286-287:
Since fgf3 is greatly upregulated in r4 in double mutants, there should be reduced neurogenesis in this region by 44 hpf. Is this the case?

Answer: As observed above, etv5b expression occurs broadly in the plzf mutant hindbrain, consistent with the broad Fgf activation and inhibition of neurogenesis that we see. This may reflect diffusion of Fgf3. We have not been able to measure the number of mature neurons accurately but do find a good correlation between Fgf pathway activation and ongoing neurogenesis detected by neurod4 expression. Thus, there is reduced neurogenesis at 30 hpf when fgf3 expression still occurs, but not at 44 hpf when fgf3 expression has been almost completely downregulated in the plzf mutants.

Lines 295-334:
While this section presents nice data on inner ear patterning defects in double mutants, it is not relevant to the main findings of the report or its significance. This section should be shortened or removed.

Answer: We have shortened this section.

Lines 336-485:
The discussion section is too long for the amount of functional data presented and the significance of the findings. Sections on comparative function of Plzf in amniotes and fish, and on Plzf in neural progenitors should be greatly truncated.

Answer: We have shortened these sections.

Lines 425-428:
This statement suggests that the effect on neurogenesis in double mutants could be non-autonomous. Such an experiment would be worthwhile and insightful and greatly strengthen this report.

Answer: Due to time constraints we have not been able to test cell autonomy, and have focussed on the plzf gain of function and fgf3 knockdown experiments to firm up the relationship between plzf and fgf3.

Minor Points

Lines 200-202, 206-208:
The phenotype at 30 hpf could result from an earlier patterning defect. Although Fig. 5 shows fgf3 expression in r4 in mutants, it is important to better document normal hindbrain patterning at 14-18 hpf in double mutants using rhombomere markers like hoxa2, egr2 and mafB. Related to this, based on extensive Plzf expression at 14-16 hpf (and perhaps at earlier stages) shown in Fig. 1, can you state definitively that there is no early role for plzf genes in hindbrain development?

Answer: We now include data showing that there is no change in hoxb1a or hoxb2 expression in plzf mutants. The organisation and amount of neurogenesis is similar in all segments during normal

© 2023. Published by The Company of Biologists under the terms of the Creative Commons Attribution License (https://creativecommons.org/licenses/by/4.0/).

development, so a change in antero-posterior patterning of segments would not account for the disrupted patterning and reduction in neurogenesis that we observe in plzf mutants.

Line 208:
While the procedure for generating the plots in Figs. 3I and S2 is described in the Methods section, it is unclear. The “center of rhombomere 2” is defined as AP position = 0. What is the “center of r2”? The values seem to be measures of the total signal in a rectangular area and not a line scan. It would be very helpful to place a schematic panel describing the measurement in Fig. S2 (and cite in Methods).

Answer: We have provided further clarification of the profile plot extraction procedure in the Methods. The reviewer is correct to point out that the line scan measures the grey value in a rectangular area, since the width of the line is set at >1. We have added two panels to illustrate this in Fig. S3.

Lines 211-212:
Is the spatial pattern of neurogenesis disrupted in mutants due to an increase in neuron number or due to more scattering of neurons into rhombomere centers? Counts of neurons in the two conditions would help address this.

Answer: We have not been able to address the question of whether the number of neurons is affected. Our focus has been on ongoing neurogenesis revealed by markers of early steps of the neurogenic cascade (neurog1, neurod4). The line scans of HCR data suggest that neurogenesis is no longer strongly patterned (i.e. now with little difference between segment centres and adjacent to boundaries) and at lower levels in the plzf mutants.

Lines 227-229, 290-291:
While the texts suggest that the anterior hindbrain is relatively unaffected in mutants, Figs. 3E and 4J indicate otherwise. In situs with rhombomere markers at 14-18 hpf (see earlier point) can address this issue definitively.

Answer: We agree that these statements were not fully supported by the data and have removed them from the text.

Lines 399-402:
Plzf expression is not rhombomere-specific in zebrafish between 14-24 hpf (Figs. 1 and 2), maybe only at 44 hpf (Fig. 2). Similarly, fgf3 expression is not anticorrelated with Plzf expression at 14-18 hpf, where high levels of fgf3 persist in the presence of high Plzf expression (Figs. 1 and 4). Please rewrite these sentences.

Answer: We have rewritten or removed these sentences to clarify them.

Second decision letter

MS ID#: DEVELOP/2022/201319

MS TITLE: Zbtb16 mediates a switch between Fgf signalling regimes in the developing hindbrain

AUTHORS: Sami Leino, Sean C.J. Constable, Andrea Streit, and David G. Wilkinson

I have now received all the referees' reports on the above manuscript, and have reached a decision. The referees' comments are appended below, or you can access them online: please go to BenchPress and click on the 'Manuscripts with Decisions' queue in the Author Area.

© 2023. Published by The Company of Biologists under the terms of the Creative Commons Attribution License (https://creativecommons.org/licenses/by/4.0/).

As you will see, while referee 2 considers the study now appropriate for Development, the other two reviewers still have concerns about some aspects of the data and the conclusions you can draw from the data. Consequently, further revision will be needed before we can consider publication.

Please attend to all of the reviewers' comments and ensure that you clearly highlight all changes made in the revised manuscript. Please avoid using 'Tracked changes' in Word files as these are lost in PDF conversion. I should be grateful if you would also provide a point-by-point response detailing how you have dealt with the points raised by the reviewers in the 'Response to Reviewers' box. If you do not agree with any of their criticisms or suggestions please explain clearly why this is so.

Reviewer 1

Advance summary and potential significance to field

Listed in my letter.

Comments for the author

The revised manuscript has been significantly improved with the addition of new experiments and explanations. Three issues remain to be addressed before granting publication:

1. In the revised figure 6, the authors rescued the expanded ETV5 expression in the zbtb16-nulls by injecting FGF3-MOs. Yet, in their response letter, the authors stated that this rescue was not sufficient to revert the aberrant-neurogenesis patterns. This is concerning, since although the manuscript shows that in mutants lacking functional zbtb16, FGF3 expression persists and the spatial pattern of neurogenesis are disrupted, these two results are circumstantial while a direct link that ties these two phenotypes together is missing. Possible experiments to validate the link between persistent expression FGF3 and perturbed neurogenic zones would be to cross the double zbtb16-null with FGF3-null, or to ectopically express FGF3 to phenocopy the disorganized neurogenesis. If these explements cannot be done, toning-down the conclusion of the study is required, such that it supports only the presented data and not interpreted beyond.
2. The Myc- zbtb16 experiment in figure 7 is an important addition.
Yet, the selected embryo (Fig.7D) is of very poor quality. I wonder why this is the case since 55% of embryo (out of 188) are mentioned to show a decreased expression of FGF3 in the injected side.
3. The authors didn't present any data regarding zbtb16b-nulls, as opposed to zbtb16a-nulls and zbtb16a&b-double nulls. This could have been a very informative control. As phenotypes don't always appear as predicted by single or double loss of paralogue genes (*Jakutis and Stainier, Genotype-Phenotype Relationships in the Context of Transcriptional Adaptation and Genetic Robustness. Annu Rev Genet. 2021 Nov 23;55:71-91*), the fact that the zbtb16b-/- embryos were not studies has to explained in the text.

Reviewer 2

Advance summary and potential significance to field

This paper describes a convincing series of experiments showing how ztb16a/b genes in zebrafish regulate Fgf signaling during successive stages of hindbrain development. I outlined the main conclusions and potential significance in my previous review. I can only add the the paper is stronger and more complete now elucidating a new mechanism controlling the transition from segmental patterning to spatially coordinated neurogenesis.

Comments for the author

The reviewers have done a very good job in addressing my previous criticisms. I have no criticisms to offer regarding the revised manuscript.

© 2023. Published by The Company of Biologists under the terms of the Creative Commons Attribution License (https://creativecommons.org/licenses/by/4.0/).

Reviewer 3

Advance summary and potential significance to field

The zinc finger Zbtb16 proteins mediate a switch in Fgf signaling regimes in the zebrafish hindbrain. Its significance remains unclear.

Comments for the author

In this revised submission, the authors have attempted to address substantial concerns raised in previous reviews. In particular, they have provided additional expression data for zbtb16a and b genes and performed two new manipulations (fgf3 MO and zbtb16a GOF) to strengthen their conclusion that zbtb16 genes are mediating a switch in fgf signaling regimes in the posterior (caudal to r4) hindbrain. The manuscript has been improved by substantially reorganizing the data and shortening the discussion.
Switching signaling regimes represents an important developmental strategy and the zbtbt16 genes may be mediating such a switch in fgf signaling in the zebrafish hindbrain. The fgf3 expression defect in double mutants is striking (Fig. 5). However, the neurogenesis phenotypes (et5vb and neurod4) are subtle and variable (Figs. 4 and 6), questioning the significance and broader impact of these findings.

Major Points
1) In Fig. 1, the whole mount (A) shows a sharp reduction poster to r4 (?) while the confocal slice (C) does not. The apparent reduction in Zbtbt16 expression posteriorly is central to the findings. How many samples do these images represent?
2) The order of Figures S2 and S3 should be switched to match their appearance in the Results section.
3) Figs. S4 and S5 are switched.

Minor Points:
Lines 349-351 are confusing. Do the authors mean that the control embryos have functional zbtbt16b but lack expression?
Lines 386-389: Why doesn't fgf3 MO extinguish etv5b expression in rhombomere centers? Are they fgf20-dependent?

Second revision

Author response to reviewers' comments

Reviewer 1
The revised manuscript has been significantly improved with the addition of new experiments and explanations. Three issues remain to be addressed before granting publication:

1. In the revised figure 6, the authors rescued the expanded ETV5 expression in the zbtb16-nulls by injecting FGF3-MOs. Yet, in their response letter, the authors stated that this rescue was not sufficient to revert the aberrant-neurogenesis patterns. This is concerning, since although the manuscript shows that in mutants lacking functional zbtb16, FGF3 expression persists and the spatial pattern of neurogenesis are disrupted, these two results are circumstantial while a direct link that ties these two phenotypes together is missing. Possible experiments to validate the link between persistent expression FGF3 and perturbed neurogenic zones would be to cross the double zbtb16-null with FGF3-null, or to ectopically express FGF3 to phenocopy the disorganized neurogenesis. If these explements cannot be done, toning-down the conclusion of the study is required, such that it supports only the presented data and not interpreted beyond.

Answer: We agree that complete loss of fgf3 function in zbtb16 double homozygotes and/or transgenic ectopic expression would provide a more direct link between fgf3 and the inhibition of

© 2023. Published by The Company of Biologists under the terms of the Creative Commons Attribution License (https://creativecommons.org/licenses/by/4.0/).

neurogenesis. Nevertheless, our findings provide strong evidence that ectopic Fgf3 signalling inhibits neurogenesis. The alternative possibilities – that ectopic Fgf3 signalling promotes or has no effect on neurogenesis - are not compatible with our findings.

To summarise, the findings that support our interpretations are as follows: (i) Loss of zbtb16 function leads to ectopic Fgf signalling (etv5b expression) at later stages; (ii) The ectopic Fgf signalling is at least in part due to failure to downregulate segmental fgf3 expression; (iii) Fgf signalling is inhibitory to neurogenesis in the zebrafish hindbrain, as shown previously by loss of function studies; (iv) Consistent with this, in the current study we find lower neurogenesis in sites of ectopic etv5b expression, and neurogenesis is increased when ectopic etv5b expression has been downregulated at 44 hpf. This provides evidence for ectopic fgf3 signalling inhibiting neurogenesis and is inconsistent with fgf3 promoting or having no effect on neurogenesis in the zebrafish hindbrain.

It does remain possible that an unidentified mechanism links zbtb16 loss of function to the decrease in neurogenesis in parallel to altered Fgf3 signalling. Such a mechanism would need to be consistent with our finding that zbtb16 is required prior to 24 hpf to enable the correct amount of neurogenesis at later stages. This can be explained by failure to downregulate an inhibitory factor. The possibility that another mechanism acts downstream of zbtb16 to inhibit neurogenesis is now mentioned (lines 554-558).

2. The Myc- zbtb16 experiment in figure 7 is an important addition. Yet, the selected embryo (Fig.7D) is of very poor quality. I wonder why this is the case since 55% of embryo (out of 188) are mentioned to show a decreased expression of FGF3 in the injected side.

Answer: All of the embryos in which zbtb16 is overexpressed have morphological changes on the injected side, which we now mention (lines 405-406). This is not unexpected as zbtb16 overexpression likely has multiple effects, which previous studies predict may include changes in cell proliferation.

3. The authors didn't present any data regarding zbtb16b-nulls, as opposed to zbtb16a-nulls and zbtb16a&b-double nulls. This could have been a very informative control. As phenotypes don't always appear as predicted by single or double loss of paralogue genes (Jakutis and Stainier, Genotype-Phenotype Relationships in the Context of Transcriptional Adaptation and Genetic Robustness. Annu Rev Genet. 2021 Nov 23;55:71-91), the fact that the zbtb16b-/- embryos were not studies has to explained in the text.

Answer: We have studied zbtb16b nulls: these are the sibling controls in the crosses used to generate double homozygous zbtb16a: zbtb16b mutants. As described in the manuscript, we generate double homozygous mutants by in-crossing heterozygous zbtb16a: homozygous zbtb16b zebrafish. The mutant phenotype described is only seen in double homozygous embryos, not in embryos of single homozygous mutants of zbtb16a or zbtb16b. We have now added text to reinforce this point (lines 247, 277-279, 568-569).

Reviewer 3
Major Points
1) In Fig. 1, the whole mount (A) shows a sharp reduction poster to r4 (?) while the confocal slice (C) does not. The apparent reduction in Zbtbt16 expression posteriorly is central to the findings. How many samples do these images represent?

Answer: As described in the text, the sharp reduction in zbtb16 expression seen in panel A is at a border in the anterior hindbrain. It cannot be at the level of r4, as this is inconsistent with the position of the otic placode which adjacent to r4 (labelled in panels A and C). The slice in panel C reveals the border to be at r1, and we have added text to reinforce this point (lines 155-157). The images represent 5 or more samples which we now mention.

2) The order of Figures S2 and S3 should be switched to match their appearance in the Results section.

Answer: Many thanks, we have now switched the order in the main text.

© 2023. Published by The Company of Biologists under the terms of the Creative Commons Attribution License (https://creativecommons.org/licenses/by/4.0/).

3) Figs. S4 and S5 are switched.

Answer: Many thanks, we have corrected the order in the Supplemental Figure file.

Minor Points:
Lines 349-351 are confusing. Do the authors mean that the control embryos have functional zbtbt16b but lack expression?

Answer: The sibling control embryos lack functional zbtb16b. This is because we generate double homozygous zbtb16a: zbtb16b embryos by in-crossing zbtb16a heterozygous: zbtb16b homozygous zebrafish. The zbtb16b homozygous mutant siblings are an appropriate control as they are indistinguishable from wild type embryos. We have added text to reinforce this point (lines 247, 277-279, 568-569).

Lines 386-389: Why doesn't fgf3 MO extinguish etv5b expression in rhombomere centers? Are they fgf20-dependent?

Answer: We have added text and a reference to mention here that etv5b expression in segment centres is mediated by fgf20 (lines 390-391). This point is also made elsewhere in the manuscript.

Third decision letter

MS ID#: DEVELOP/2022/201319

MS TITLE: Zbtb16 mediates a switch between Fgf signalling regimes in the developing hindbrain

AUTHORS: Sami Leino, Sean C.J. Constable, Andrea Streit, and David G. Wilkinson
ARTICLE TYPE: Research Article

I am happy to tell you that I am happy with your responses to the previous round of reviews and your manuscript has been accepted for publication in Development, pending our standard ethics checks.

© 2023. Published by The Company of Biologists under the terms of the Creative Commons Attribution License (https://creativecommons.org/licenses/by/4.0/).
